# High throughput reaction screening using desorption electrospray ionization mass spectrometry†
†Electronic supplementary information (ESI) available. See DOI: 10.1039/c7sc04606e


**DOI:** 10.1039/c7sc04606e

**Published:** 2018-01-04

**Authors:** Michael Wleklinski, Bradley P. Loren, Christina R. Ferreira, Zinia Jaman, Larisa Avramova, Tiago J. P. Sobreira, David H. Thompson, R. Graham Cooks

**Affiliations:** a Department of Chemistry , Purdue University , West Lafayette , IN 47907 , USA . Email: cooks@purdue.edu

## Abstract

We report the high throughput analysis of reaction mixture arrays using methods and data handling routines that were originally developed for biological tissue imaging. Desorption electrospray ionization (DESI) mass spectrometry (MS) is applied in a continuous on-line process at rates that approach 10^4^ reactions per h at area densities of up to 1 spot per mm^2^ (6144 spots per standard microtiter plate) with the sprayer moving at *ca.* 10^4^ microns per s. Data are analyzed automatically by MS using in-house software to create ion images of selected reagents and products as intensity plots in standard array format. Amine alkylation reactions were used to optimize the system performance on PTFE membrane substrates using methanol as the DESI spray/analysis solvent. Reaction times can be <100 μs when reaction acceleration occurs in microdroplets, enabling the rapid screening of processes like *N*-alkylation and Suzuki coupling reactions as reported herein. Products and by-products were confirmed by on-line MS/MS upon rescanning of the array.

## Abstract 2

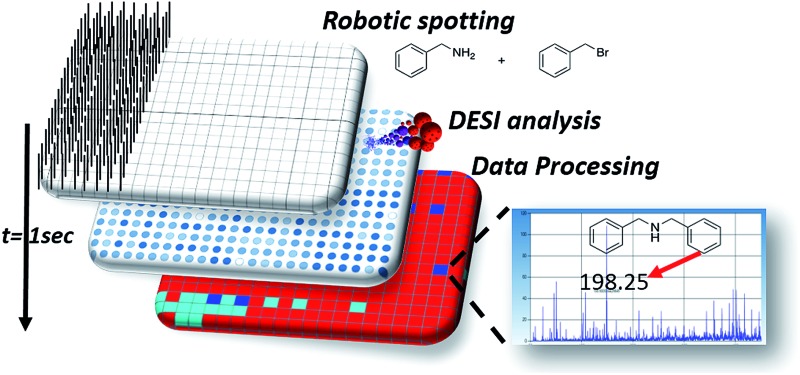
We report the high throughput analysis of reaction mixture arrays using methods and data handling routines that were originally developed for biological tissue imaging.

## Introduction

Pharmaceutical discovery depends on screening large numbers (“libraries”) of compounds against biological targets of therapeutic interest. In some cases, this involves evaluation of large collections of natural products and their derivatives for biological activity.^1^–^4^ Current estimates suggest that there are more than 10^60^ drug-like molecules of pharmaceutical interest and over 10^7^ possible reaction conditions for a single metal-catalyzed reaction used to build a drug scaffold.^5^–^8^ This reality underscores the need for rapid reaction screening and optimization, particularly with the introduction of automated synthesis techniques and the use of combinatorial methods to generate large numbers of closely related compounds.^9^ An illustration of the current bottleneck is given by the fact that compound library sizes of a million are common, requiring two years of continuous operation even at conventional UPLC sampling rates of 1 min per sample. Clearly, multiplexing of the sample transfer, reaction, and/or product analysis is essential to remove this bottleneck. The first two of these steps are readily multiplexed using microtiter well plate formats. The analysis step can also be multiplexed in a variety of ways, including fluorescence analysis with 2D imaging detection. Unfortunately, fluorescence techniques require orthogonal labels and have limited molecular specificity, while label-free approaches like mass spectrometry (MS) are serial analytical tools.^10^ State of the art LC-MS high throughput methods require roughly 10 s per sample.

In this paper, we describe an MS method that analyses product outcomes in reaction droplets within about 1 s. If proven to be sufficiently rugged and reliable, this approach will reduce the analysis time for 10^5^ reactions from 2 months to about a day. Furthermore, the traditional process of route design, reaction optimization, and scaling can take years to successfully implement for many target compounds. The use of high throughput experimental methods to guide scalable syntheses such as continuous-flow reactors has the potential to drastically improve the speed at which an optimized process can be achieved at scale. Accelerated reactions in microdroplets have been previously shown capable of guiding optimization of continuous-flow syntheses of multiple targets,^11^ including diphenhydramine,^12^ atropine,^13^ and diazepam.^14^ The overall goal of this work is the development of a system that can leverage rapidly acquired information on reaction acceleration from a high throughput format and use the output data to inform downstream scaling.

The combination of rapid reaction screening and reaction product analysis described here is based on two well-established analytical technologies: (i) the use of desorption electrospray ionization (DESI) in surface analysis^15^–^17^ and (ii) the occurrence of accelerated chemical reactions in microdroplets.^18^–^20^ DESI is an ambient ionization method (*i.e.*, analysis is performed on unmodified samples in the open air) using charged microdroplets to extract analytes from complex samples. It does so with ∼200 μm spatial resolution and with rapid data acquisition (typically < 1 s per spot).^21^,^22^ The combination of another ambient method, paper spray, with *in situ* analysis has also been reported.^23^

**Fig. 1:**
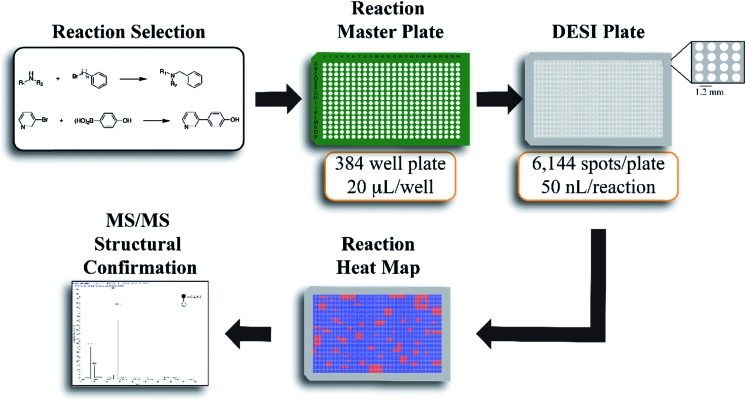
Summary of the high throughput reaction screening experiment workflow with DESI-MS. Both the master plate and the DESI plate are prepared in an automated fashion with a liquid handling robot. The DESI plate (porous PTFE) is prepared with a 50 nL pin tool.

The workflow for the automated high throughput reaction screening experiment with DESI-MS is described in Fig. 1. Upon selection of the reactions, a Beckman-Coulter Biomek FX liquid handling robot is used to prepare the reaction mixtures in a 384 well plate (20 μL per well). The liquid handling robot then transfers nL volumes of each reaction mixture to the DESI plate (porous PTFE) with a magnetic pin tool. The small spot sizes achieved by the transfer pins allow for the preparation of high density plates, which are subjected to DESI-MS immediately following their preparation. In-house software is then used to generate a reactivity heat map capable of guiding MS/MS experiments for structural confirmation of both products and byproducts.

The center-to-center reaction mixture spot distance in a 6144 array is 1125 microns, thus easily accommodating one full MS scan. The DESI-extracted analytes are released from the surface in the form of microdroplets. High throughput DESI-MS reaction screening is based, in part, on the occurrence of accelerated chemical reactions in these microdroplets.^24^–^27^ This forms the temporal basis for an experiment wherein an array of reaction mixtures (for example on a microtiter plate) is examined by rastering a DESI spray across the surface. Previous studies indicate that there is good correlation between microdroplet chemistry and more traditional batch or continuous chemistries.^11^–^14^ This study focuses on the development of a high throughput screening system with amine alkylation and Suzuki cross-coupling transformations to further test this expectation. In both cases, reagent screening was also evaluated.

**Fig. 2:**
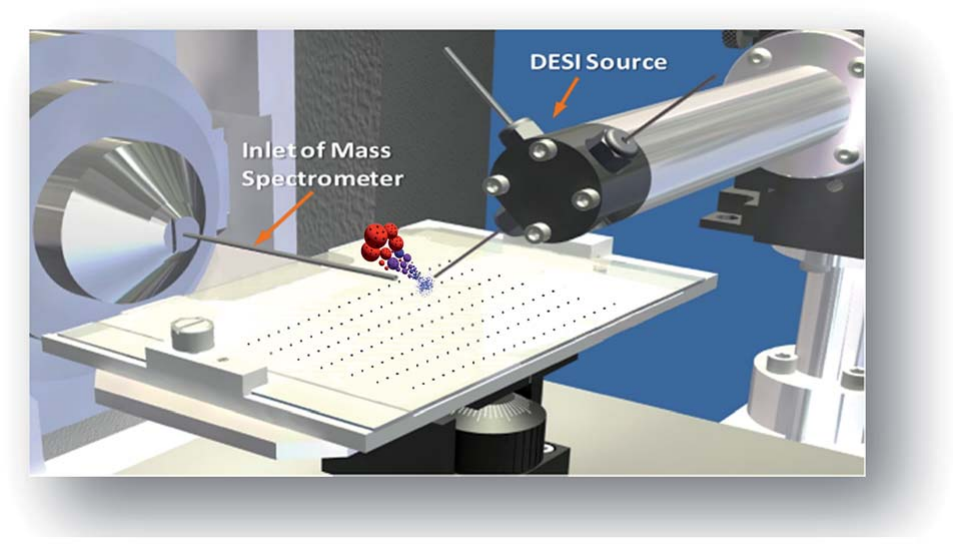
Reaction (in secondary droplets) and MS product analysis of reaction mixtures presented in microtiter plate format. Automated acquisition of DESI mass spectra occurs at a rate of 1 reaction mixture per s.

The phenomena involved in reaction screening are similar to those taking place in DESI-MS tissue imaging, so the same instrumentation and software can be used to gather and process the data. Fig. 2 illustrates the high throughput DESI analysis of an array of reaction mixtures. Note that a mixture of reagents is deposited at each well, with the DESI spray potentially accelerating both the reaction and facilitating the analysis of the reaction mixture. Earlier work^6^ on high throughput HPLC-MS analysis using the microtiter format represents an alternative approach with analysis times of 5–22 s per sample while commercial SPE MS systems, such as the RapidFire 365 system, allow rates of up to 8 s per sample.^8^ The use of DESI-MS to evaluate reactions in a high-throughput fashion suited our overall objective to develop and demonstrate a novel, fast, and reliable system for solution-phase reaction screening. The novelty of this study is to seek to extend the speed with which large numbers of microdroplet reactions can be carried out and their products analyzed.

The system was intended to provide a rapid answer to the yes/no question of whether or not a particular set of reagents produces a desired product. If a reaction occurs under DESI-MS conditions, it is considered a candidate for further investigation as a scaled-up version of the reaction for process optimization. If not, this possible route to a particular product under those conditions is discarded. In roughly the same amount of time that it takes to record data for a single product, one can collect MS data on by-products, reaction intermediates, and residual starting material in the same mass scan. Full mass spectra, therefore, were recorded on each spot of reagent mixture at a target throughput of 10 000 reactions per h. Software was written to automatically screen for ion *m*/*z* values corresponding to reagents, reaction products and simple by-products. For selected target ions, re-analysis at particular locations was performed using MS/MS data acquisition to allow product confirmation while retaining the advantages of high throughput imaging.

## Results

### *N*-Alkylation reactions

The ultimate goal of this effort is to develop screening capabilities across a broad range of reaction conditions for synthetic optimization and reaction discovery. This led us to evaluate the capability of screening different reaction classes such as *N*-alkylation and Suzuki cross-coupling given their importance in medicinal chemistry transformations.^28^

**Fig. 3:**
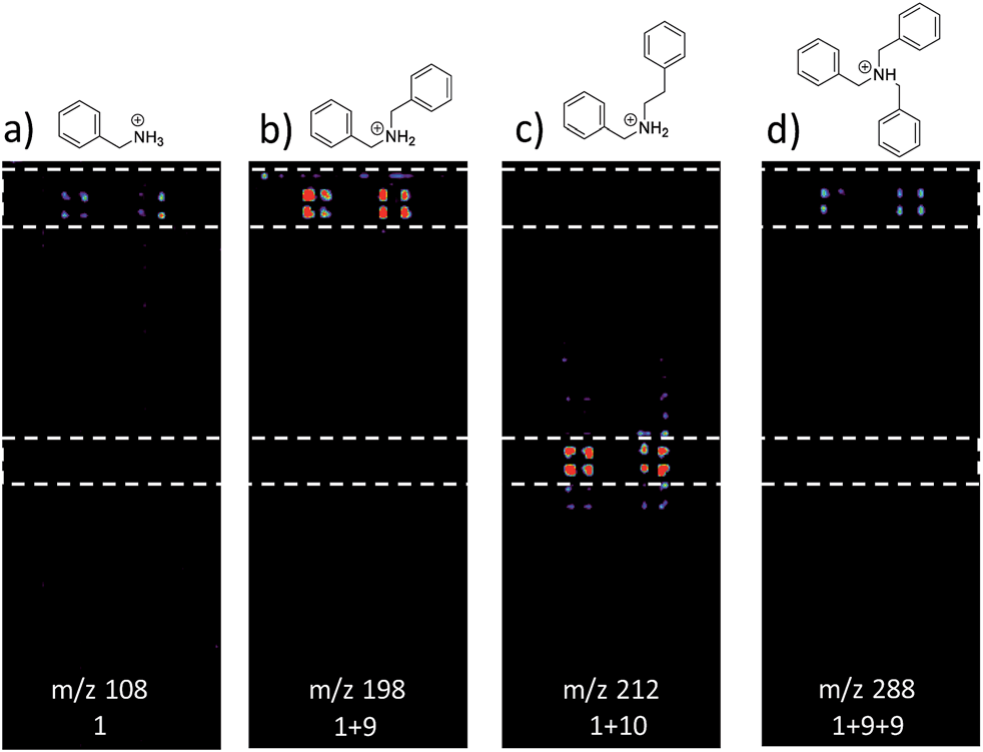
Selected ion images for reactions between amines and alkyl bromides. Images are (a) *m*/*z* 108, benzylamine; (b) *m*/*z* 198, alkylation product for reaction of **1** and **9**; (c) *m*/*z* 212, alkylation product for reaction of **1** and **10**; (d) *m*/*z* 288, double alkylation product for reaction of **2** and **9**.

**Scheme 1:**
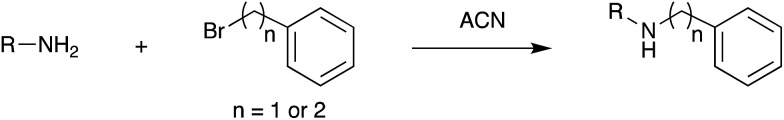
*N*-Alkylation reactions.

Fig. 3 shows selected data from an experiment wherein a set of 16 alkylation reactions (Schemes 1, S1, and S2†) was studied by spotting mixtures of eight amines (benzylamine, **1**; 1-aminohexane, **2**; *N*,*N*-dihexylamine, **3**; 2-methoxyaniline, **4**; 3-methoxyaniline, **5**; 4-methoxyaniline, **6**; piperidine, **7**; *N*-methylbenzylamine, **8**) and two alkyl bromides (benzyl bromide, **9**; 2-(bromoethyl)benzene, **10**) in a 1 : 1 molar ratio on a porous PTFE substrate at the density of a 1536-well microtiter plate. Spotting was done using a Beckman-Coulter Biomek FX liquid handling system equipped with small (typically 50 nL) slotted delivery pins. Fig. 3 illustrates the data for the reaction between **1** and **9** as well as **1** and **10**, each panel showing the location of spots which display ions corresponding to particular reagents or products. The brighter colors indicate higher ion intensity. A series of mass spectra was continuously recorded while covering the entire plate by scanning row by row at a slightly faster scan speed of 6250 μm s^–1^, but with a lower spot density and averaging 3 mass spectra per array spot and achieving a screening rate of 1284 reactions per h. Note that this time includes reaction and analysis times, but not the time required for sample preparation.

Note also that rhodamine B (*m*/*z* 443) was used as a positional reference. Significant features of the experiment are: (i) starting materials were often, but not always visible; (ii) product formed readily with **9** and much less readily with **10**; (iii) there is no evidence of an elimination reaction with **10**; (iv) double alkylation reactions are commonly observed with **9**; (v) there is little sign of sample carry-over, a desirable feature that is also true of tissue imaging by DESI-MS; and (vi) there is some variability in signal when replicates are examined. Experimental details are in the ESI† (Section 2) and results for the other reactions are detailed in Table 1. The reaction was successful for 7 out of 8 amines with benzyl bromide and 3 out of 8 amines with 2-(bromoethyl)benzene, the difference reflecting the known difference in bromoalkane reactivity.

**Table 1:** Summary of results from *N*-alkylation DESI reaction screen

Amine	Product *m*/*z*	Alkylation product?	*m*/*z*	Double alkylation product?
**Amine + Benzyl bromide**				
Benzylamine	198	✓	288	✓
Hexylamine	192	✓	282	✓
Dihexylamine	276	✓	366	✗
*o*-Anisidine	214	✓	304	✓
*m*-Anisidine	214	✓	304	✗
*p*-Anisidine	214	✓	304	✓
Piperidine	176	✓	266	✓
*N*-Benzylmethylamine	212	✓	302	✓

**Amine + 2-(bromoethyl)benzene**				
Benzylamine	212	✓	316	✗
Hexylamine	206	✓	310	✗
Dihexylamine	290	✗	394	✗
*o*-Anisidine	228	✗	332	✗
*m*-Anisidine	228	✗	332	✗
*p*-Anisidine	228	✗	332	✗
Piperidine	190	✓	294	✗
*N*-Benzylmethylamine	226	✗	330	✗

These reactions may be accelerated in the droplets, or even earlier, since the 50 nL reaction mixtures containing less than 0.5 μg of each reagent (Table S4†) may evaporate on the PTFE plate. They may occur in the master plate well for very fast reactions. In any event, the DESI screen answers affirmatively in this case the yes/no question being asked and conversion rates (CR) can be calculated in order to estimate the reaction completeness (Table S3†). Product characterization (Tables S3 and S4†) was by tandem mass spectrometry, as discussed below.

### Suzuki cross-coupling reactions

**Scheme 2:**
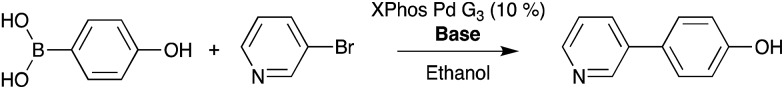
Palladium-catalyzed Suzuki cross-coupling reaction. Bases used include EtONa, EtOK, MeONa, and MeOK.

Suzuki cross-coupling between phenylboronic acid precursors and aryl halides using a commercially available palladium catalyst (XphosPD G3) was also screened (Scheme 2). All of the reactions were run in ethanol, and the effect of base on the reaction was explored with EtONa, EtOK, CH_3_ONa, and CH_3_OK using rhodamine as a fiducial marker. Reaction mixtures were prepared in a 384 well master plate (20 μL per well) and spotted onto a porous PTFE substrate using 6 nL slotted pins. All of the reaction mixtures were prepared in quadruplicate wells and the entire master plate was spotted in quadruplicate to achieve 1536 density on the final DESI plate.

**Fig. 4:**
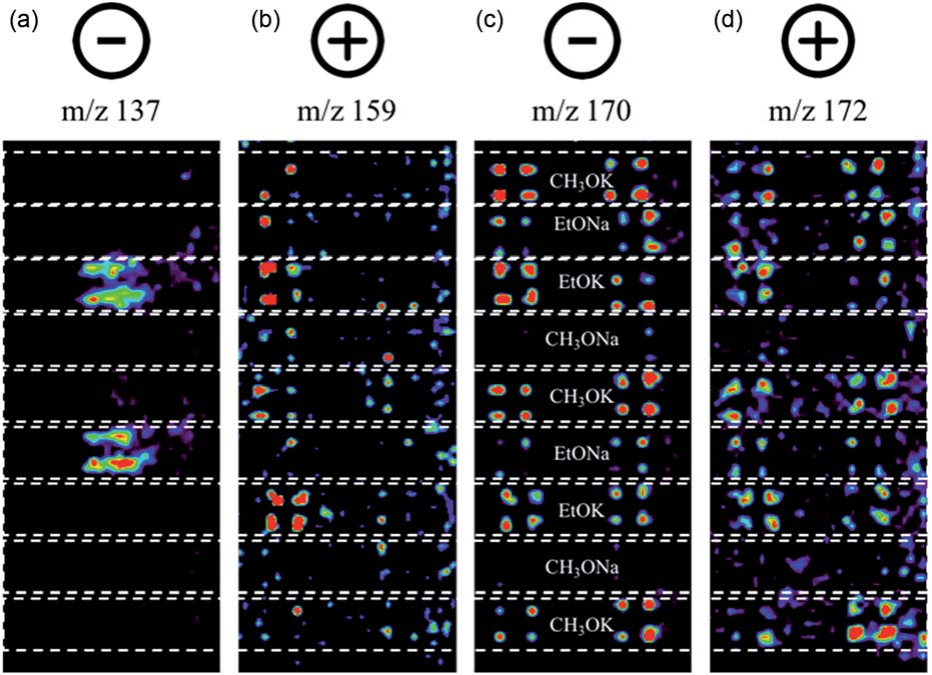
Selected ion images for reactions between **11** and **12**. Images. (a) Negative ion mode *m*/*z* 137, **11**; (b) positive ion mode *m*/*z* 159, **12**; (c) negative ion mode *m*/*z* 170, **13**; (d) positive ion mode *m*/*z* 172, **13**.

In this experiment, one sees the boronic acid starting material in negative ion mode (*m*/*z* 137), aryl bromide starting material in positive ion mode (*m*/*z* 159), and cross-coupled product in both modes (*m*/*z* 170 and 172, respectively). The reaction proceeds well in 3 of the 4 bases, namely CH_3_OK, EtOK, and EtONa (Fig. 4 and S8, Table S6†).

### Optimization of pinning and DESI analysis

Porous PTFE has been chosen as a reaction substrate due to the high ion intensities for the reaction product, low background and high chemical compatibility compared to non-porous PTFE, Nylon, cellulose, and DMF (data not shown). A standard porous PTFE plate containing a single mine alkylation reaction mixture (benzylamine, **1** and benzyl bromide, **9**) was used to develop an understanding of the experiment, including (i) achievable number of reactions per h and (ii) the effects of mechanical instabilities on data quality. Considering these points in turn: (i) under the solvent and instrument setting conditions used for the experiments at 6144-well microtiter plate spot density, the fastest analysis speed achieved was 6004 reactions per h with a lateral spray velocity of 8333 μm s^–1^ (Fig. S10†). (ii) Distinct spots can be deposited at densities as high as 24 457 per standard plate, as confirmed by fluorescence (Fig. S11†), but the spots blur at DESI analysis speeds above 10 000 reaction mixtures per h.

**Fig. 5:**
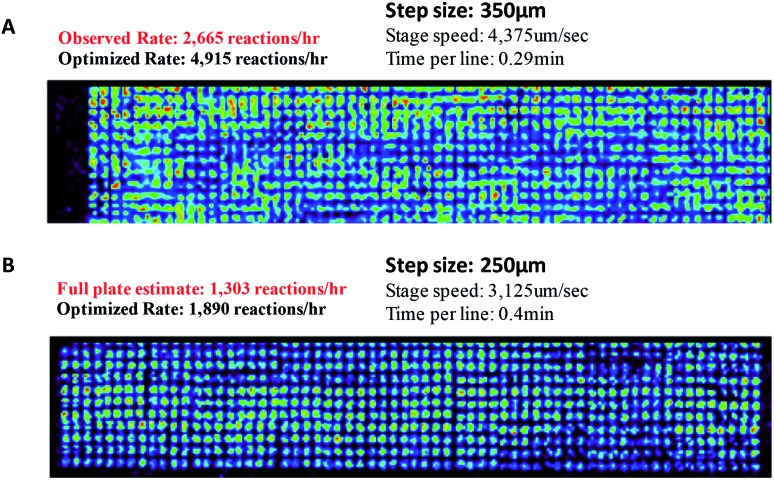
DESI analysis of the reaction between benzylamine (**1**) and benzyl bromide (**9**) at different speeds of the moving stage.

Furthermore, it is notable that there is variation in intensity across the plate surface as well as small variations in spot placement. Two possible causes for the decrease in signal intensity are that the pins do not deliver the appropriate volume of solution to the substrate and that DESI-MS analysis performance drops due to surface roughness or non-planarity. Fluorescence imaging and DESI-MS were used to explore these factors. Pins used in these experiments differed in diameter, shape, length, and prior use history. Pins (6 nL) that were heavily used in earlier screening experiments produced spots with variation in fluorescence intensity, indicating the inability to deliver a constant volume of sample to the substrate. When thicker pins calibrated to 50 nL were used, the intensity was relatively uniform. With optimized equipment and spotting procedures, the variation in signal intensity is low. Fig. 5 details an experiment in which 50 nL pins were used to deposit a single reaction mixture of **1** and **9** at 6144 density. The reactions were screened at varying speeds of the moving stage. We found that data quality increases with decreasing speed of the moving stage. The optimized rate was calculated by eliminating the time for line transitions on the moving stage. It is noteworthy that even at four times higher density (6144 *vs.* 1536), DESI-MS showed similar performance to fluorescence (compare Fig. S9–S11†) while giving much more information.

### Point-to-point DESI MS/MS

**Fig. 6:**
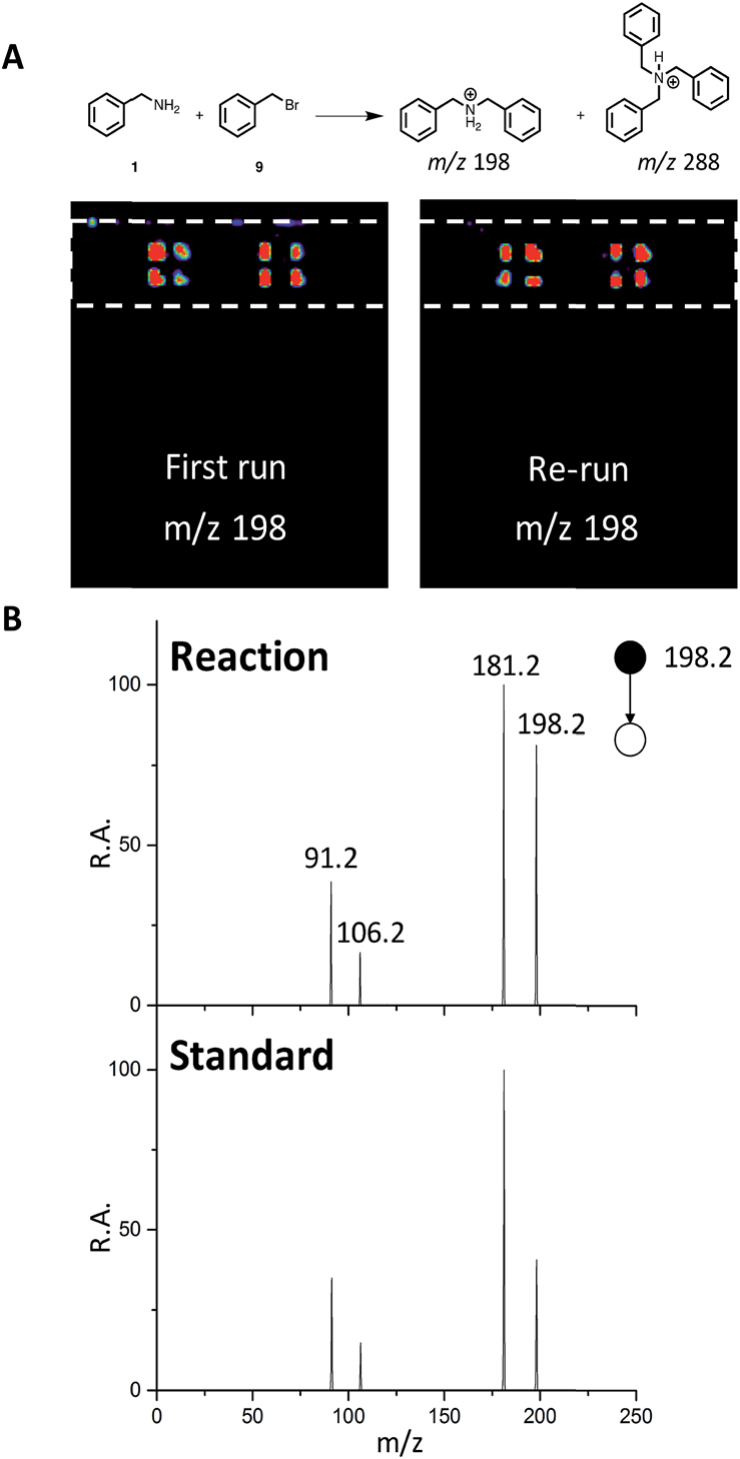
(A) DESI-MS images for reaction between **1** and **9** for the first run and a re-run of the same plate. (B) MS/MS of reaction product (*m*/*z* 198) and a dibenzylamine standard.

We next sought to develop the capability for rapid structural confirmation by MS/MS immediately following the DESI reaction screen on the same substrate. To accomplish this, we first needed to determine whether a plate could be re-analyzed without blurring the sample on the surface or causing significant depletion of material. This was done for the entire *N*-alkylation plate (Fig. S2–S6). The reaction between **1** and **9** is shown in Fig. 6A. In all eight replicates shown, there is clearly no significant blurring or sample depletion. With this established, we were then able to confidently move forward with the development of an MS/MS methodology on the same reaction plate. The data shown in Fig. 6B utilizes the point-to-point feature of the DESI system. Once the reaction hits are determined, a spreadsheet is uploaded into the commercial 2D DESI platform software (Prosolia Inc. Indianapolis, IN, US) with their *x* and *y* positions. The DESI sprayer then oscillates four times over each location while the mass spectrometer is collecting the desired MS/MS spectra for the products and by-products. This methodology is detailed in Fig. S7 and the data is summarized in Tables S4 and S5.†

This was done for all of the *N*-alkylation reactions with **9** for both the single alkylation product and the double alkylation byproduct. The mass spectrum for the reaction between **1** and **9** (Fig. S12A†) shows several products, including the target dibenzylamine *m*/*z* 198, confirmed as such by on-line by MS/MS and comparison with a standard (Fig. 6). The MS/MS shows elimination of NH_3_ to give the *o*-benzyl benzyl cation (*m*/*z* 181) through benzyl rearrangement. The ion pair *m*/*z* 475/477 (Fig. S17†) is a brominated by-product while the ion *m*/*z* 288, corresponds to a by-product, assigned after independent synthesis and MS/MS characterization as tribenzylamine (Fig. S14–S17; Scheme S3†).

Reanalysis of a plate typically gives high quality data in both MS and MS/MS modes (ESI, Fig. S18 and S19†). Reaction product structural analysis can be interrogated beyond MS^2^ and this capability will be incorporated in the ongoing automation of mass spectra interpretation. As an example, the reaction of dihexylamine and benzyl bromide has been used to acquire MS^3^ and MS^5^ data directly from the PTFE plate spotted at 6144 density. The alkylation product of *m*/*z* 276, yielded the most intense fragment of *m*/*z* 184, which has been further isolated for examination by MS^3^ (Fig. S19†). For the double alkylation product of *m*/*z* 366, after MS^2^ the most intense fragment is *m*/*z* 274 due the loss of alkene (C_7_H_8_), which fragments further (MS^3^) to yield *m*/*z* 204 due to another alkene loss (C_5_H_10_). MS^4^ of *m*/*z* 204 was followed by another alkene loss (C_5_H_10_) to yield *m*/*z* 134, which was intense enough to be isolated for MS^5^ (Fig. S20†). The ion C_7_H_7_ (*m*/*z* 91) is observed as a fragment at MS^3^ through MS^5^ stages. MS^3^ of *m*/*z* 276 and MS^5^ of *m*/*z* 366 in point-analysis experiments provided data of similar quality (data not shown).

### Automation of MS analysis

**Fig. 7:**
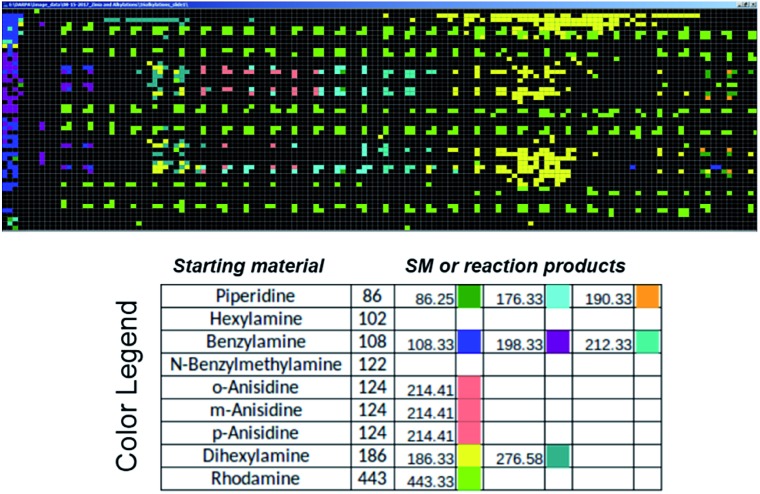
Output of the software intended to automatically perform data analysis during high-throughput reaction screening by DESI-MS. The output depicted shows starting materials (SM) and reaction products in different colors for the sixteen different *N*-alkylation reactions described in Schemes S1 and S2,† and for which ion images have been recorded using the imaging software BioMAP in Fig. S2–S6.†

Commercial software (Prosolia, Inc.) was used to operate the DESI system. Data were acquired using a commercial instrument (Thermo, LTQ) and converted into images using commercial software (Prosolia, Inc.). The total ion current plots simply represent images of the array. The question of what specific data to examine in the high spatial resolution full mass spectral data space is addressed using in-house software to automate MS data analysis. Fig. 7 shows application of this methodology to plotting starting materials and potential reaction products of all the alkylation reactions in a compact format. One function of the in-house software is to automatically search the captured data for *m*/*z* values that correspond to reactants and products to generate a yes/no report. Known intermediates, products and by-products can also be identified by rules-based input. Another function is to display the output as an *x*, *y* image of this search as shown in Fig. 7. A third feature of the software is that it displays the mass spectrum at any coordinate position upon clicking the image (as shown in the TOC). Computational approaches to the more challenging problem of identification of unknowns and by-products are being evaluated in an extension of the program using MS/MS data, taken either on-line or during a repeat scan.

## Conclusions

Initial development of this DESI-MS screening methodology (i) holds promise for high speed reaction screening while using minute amounts of chemicals/(low μg level); (ii) provides information valuable in scaled-up synthesis including solvent and pH optimization; and (iii) gives good qualitative reproducibility although this suffers at the highest speeds. Our results indicate that the engineering requirements for the highest speed screens will be demanding in terms of quality of reaction mixture spotting, the planarity of the plate, accurate sample positioning on the plate and spray reproducibility. Development of automation and integration methodology for high-throughput reaction screening by DESI-MS is underway. Software to allow automated and near real-time analysis of MS and MS/MS data is under development. We show initial data obtained using this tool (Fig. 7) but do not explore details in this manuscript. Innovations in fluid handling are under development in order to allow preparation of reaction mixtures at speeds to match the time taken for reaction and analysis. The strategy of using accelerated reactions has already been adapted to synthetic route screening and is currently being extended to optimization. As shown, it allows structural confirmation and identification of targets using MS/MS. Translation of results from high throughput DESI reaction screens to continuous-flow reactions is underway.

## Experimental methods

### Preparation of reaction mixtures

All chemicals and reagents were purchased from Sigma Aldrich and used without further purification. Stock solutions (0.1 M) were prepared in appropriate solvents as indicated. Master 384-well microtiter plates were prepared either by hand pipetting appropriate mixtures of chemicals or by robotic pipetting with a Biomek FX liquid handling system. Once the master plate was prepared, a 384-pin tool from V&P Scientific, Inc. was used to deliver mixtures from the master plate to the DESI-MS substrate. Transfer was repeated multiple times in order to generate the desired titer plate density (1536-well, 6144-well, *etc.*). Details for each individual experiment are provided in the ESI.†

### DESI-MS substrates

A variety of DESI-MS substrates were tested including regenerated cellulose (Membrane Filtration Products, Inc.), PTFE (EMD, Millipore Fluoropore, Saint-Gobain), glass fiber (Foxx Life Sciences), and nylon (Foxx Life Sciences). The substrates were cut with scissors and adhered to glass slides using a spray adhesive (Scotch Spray mount). No signs of interference from the glue was observed.

### Mass spectrometer parameters

A commercial DESI source from Prosolia, Inc. was used for the *N*-alkylation experiments and MS/MS analysis. A homebuilt DESI ion source similar to the commercial source available from Prosolia, Inc. was used for the Suzuki experiments. A Thermo LTQ linear ion trap was used to acquire data. DESI-MS experiments were performed in both positive and negative ion mode over the range *m*/*z* 50–500. The ion source parameters are detailed in the ESI.† Membrane substrates were scanned horizontally at a rate between 5000 and 10 000 μm s^–1^ using vertical steps of 500 μm. The MS injection time was set so as to produce square pixels of 500 by 500 μm. Data were first converted using a in-house software into a format compatible with Biomap. Biomap (freeware, ; https://ms-imaging.org/wp/biomap/) was used to generate the selected ion images by choosing appropriate ion thresholds.

## Conflicts of interest

There are no conflicts to declare.

## Supplementary Material

Supplementary information
