# Supplementary material for: High throughput reaction screening using desorption electrospray ionization mass spectrometry
Source: Chem Sci. 2018 Jan 4;9(6):1647–53. doi: 10.1039/c7sc04606e (PMC5887808; doi:10.1039/c7sc04606e)
Supplement: Supplementary file 1 [file SC-009-C7SC04606E-s001.pdf]

Electronic Supplementary Material (ESI) for Chemical Science.
This journal is © The Royal Society of Chemistry 2018

SUPPORTING INFORMATION

High Throughput Reaction Screening using Desorption Electrospray Ionization Mass Spectrometry

Michael Wleklinski, Bradley P. Loren, Christina R. Ferreira, Zinia Jaman, Larisa Avramova, Tiago Sobreira, David H. Thompson and R. Graham Cooks

Department of Chemistry
Purdue University
West Lafayette, IN 47907

Contents

1. **General DESI-MS Analysis Procedure……………………………………………………………………….p. 2**
2. **Alkylation reaction between 8 amines and 2 alkyl bromides………………………………………..p. 2**
3. **Suzuki cross coupling ……………………………………………………………………………………………….p. 14**
4. **Ultrahigh density spotting experiment……………………………………………………………………..p. 15**
5. **Typical MS spectra…………………………………………………………………………………………………….p.18**
6. **Product identification by MS/MS……………………………………………………………………………….p.19**
7. **On-line MS/MS by re-analyzing selected pixels…………………………………………………………..p.24**
8. **Structural elucidation of reaction products by MS…………………………………………………….p.25**

1. General DESI-MS Analysis Procedure

DESI-MS was performed using a homebuilt ionization source utilizing the following typical conditions. We consistently evaluated selected alkylation reactions using different solvents for the DESI spray. Pure methanol the yielded best results and it has been used as the analysis solvent at the flow rate of 3-8 µl/min. Sheath gas: N_2, pressure 120 psi. Injection time: 10 -15 ms for 1 microscan; mass scan time 80 ms; DESI moving stage speed: 6,250 µm/sec. Mass range: typically *m/z* 50-500. Analysis area: typically 75,500 µm by 25,500 µm (size of a standard microtiter plate); x and y resolution 500 µm. Mass spectrometer: ion trap, model XL (Thermo Scientific, San Jose, CA). Data acquisition used the commercial Prosolia 2D DESI stage control system in combination with their Firefly software to convert data from Thermo format (.raw) to a format compatible with the BioMAP imaging freeware (.img). The reaction time is less than the time taken to record a single mass scan, viz. less than 80 msec.

The rate of DESI-MS analysis (averaging multiple mass scans per spot) was calculated using the equation:

$$Rate = \frac{\#\ of\ spots}{\Delta T_{Analysis}}\ (1)$$

where $\Delta T_{Analysis}$ is defined by

$$\Delta T_{Analysis} = \Delta T_{Line-Scan} * \#lines + \Delta T_{Reset} * \#\ lines\ (2)$$

The time for analysis of a line scan ($\Delta T_{Line\text{-}Scan}$) is defined as the length of the line divided by the stage velocity. ΔT_{Reset} is defined as the time it takes to travel to the next line start point in the image. The typical time for reset varied from 3 seconds to 20 seconds. For the rates calculated in this paper the reported rate is that which uses a 9 second return time, while the optimized rate is calculated using a 3 second return time.

2. Alkylation reactions between 8 amines and 2 alkyl bromides

Procedure for preparation of reaction plate and stamping of DESI-MS plates

Stock solutions of each amine (0.1 M in acetonitrile) and alkyl bromide (0.1 M in acetonitrile) were prepared in 1:1 ratio and dispensed into a 384 master plate resulting in a final volume of 100 µl in each well. Additionally, 100 µl of a prepared solution of Rhodamine B (25 mg/ml in acetonitrile acidified with ~10 µl of 4M HCl in dioxane) was also added to the indicated wells (Figure S1). A pin tool fitted with 50nL pins (unless otherwise indicated) was used to transfer solution from the master plate onto suitable DESI-MS substrates. Each sample well in the master plate was transferred in quadruplicate resulting in 1536 density on the microtiter plate used as the DESI-MS substrate. The freshly prepared membrane substrate was transferred to a homebuilt DESI source for analysis. The analysis speed was 6,250 µm/sec resulting in a reaction rate of 1,284 reactions/hr in this case.

Scheme S1. Structures of amines

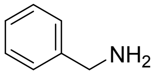

Molecular Weight: 107.16
1

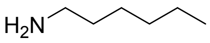

Molecular Weight: 101.19
2

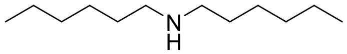

Molecular Weight: 185.36
3

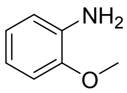

Molecular Weight: 123.16
4

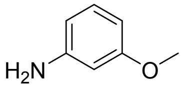

Molecular Weight: 123.16
5

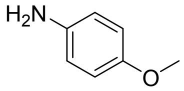

Molecular Weight: 123.16
6

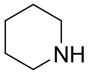

Molecular Weight: 85.15
7

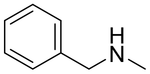

Molecular Weight: 121.18
8

Scheme S2. Structures of alkyl bromides

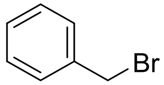

benzyl bromide
9

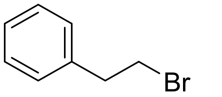

2-Phenylethylbromide
10

	1	2	3	4	5
A	RhB	1+9	RhB	1+9	RhB
B	RhB	2+9	RhB	2+9	RhB
C	RhB	3+9	RhB	3+9	RhB
D	RhB	4+9	RhB	4+9	RhB
E	RhB	5+9	RhB	5+9	RhB
F	RhB	6+9	RhB	6+9	RhB
G	RhB	7+9	RhB	7+9	RhB
H	RhB	8+9	RhB	8+9	RhB
I	RhB	1+10	RhB	1+10	RhB
J	RhB	2+10	RhB	2+10	RhB
K	RhB	3+10	RhB	3+10	RhB
L	RhB	4+10	RhB	4+10	RhB
M	RhB	5+10	RhB	5+10	RhB
N	RhB	6+10	RhB	6+10	RhB
O	RhB	7+10	RhB	7+10	RhB
P	RhB	8+10	RhB	8+10	RhB

Figure S1: Reaction solution positions in master plate for amine/alkyl bromide

Table S1 List of Components in master plate alkylation reactions

**1**	Benzylamine	**6**	p-Anisidine
**2**	Hexylamine	**7**	Piperidine
**3**	Dihexylamine	**8**	N-Benzylmethylamine
**4**	o-Anisidine	**9**	Benzyl bromide
**5**	m-Anisidine	**10**	2-Bromoethylbenzene

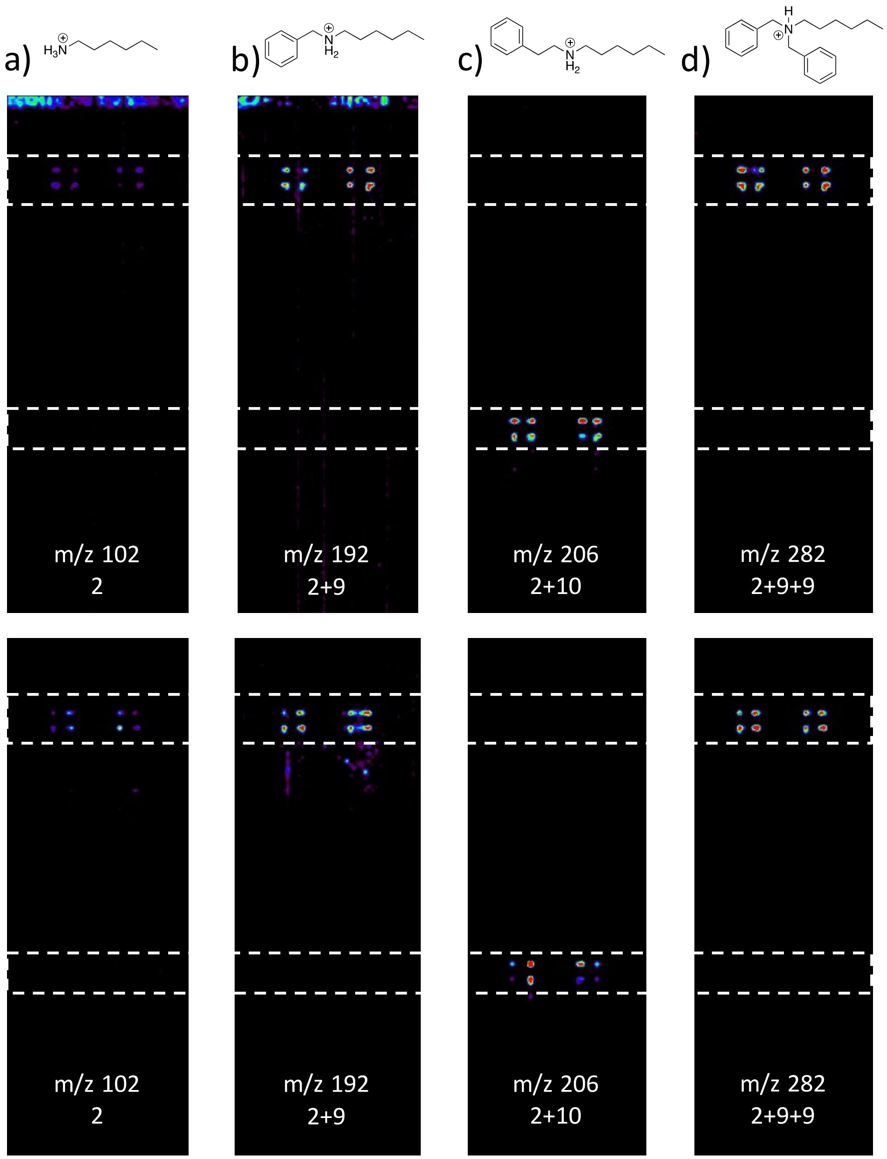

Figure S2. Selected ion images from reaction between 2+9 and 2+10. a) *m/z* 102, b) *m/z* 192, c) *m/z* 206, and d) *m/z* 282. The bottom set of images was obtained by repeating the DESI analysis on the same sample.

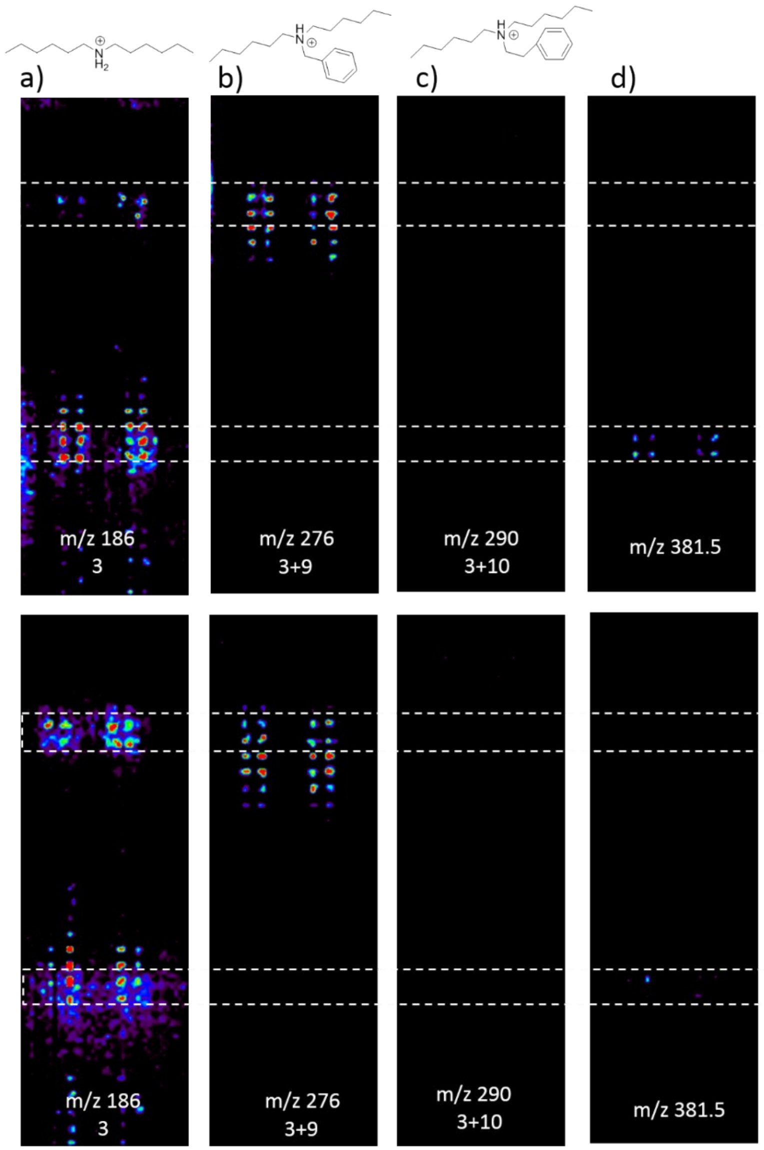

Figure S3. Selected ion images from reaction between 3+9 and 3+10. a) *m/z* 186, b) *m/z* 276, c) *m/z* 290, and d) *m/z* 381.5. The bottom set of images was obtained by repeating the DESI analysis on the same sample**.**

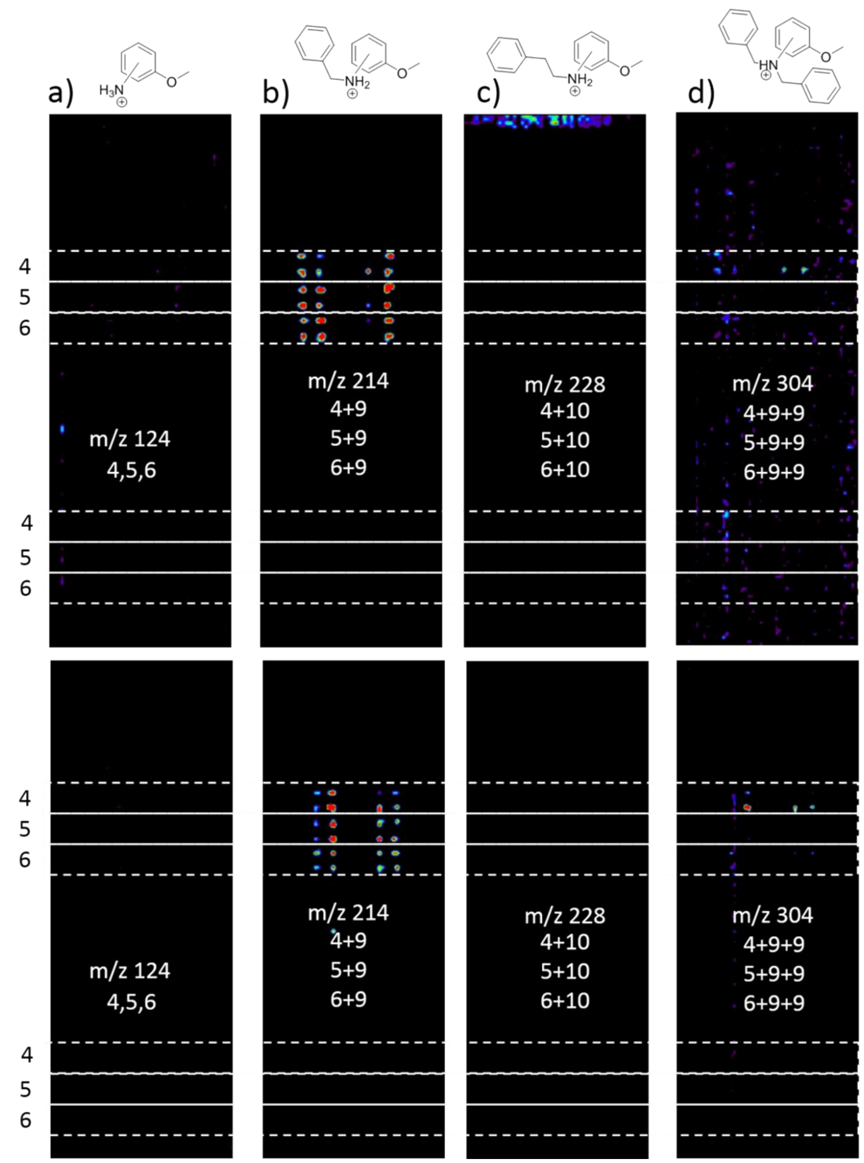

Figure S4. Selected ion images from reaction between 4+9, 5+9, 6+9 and 4+10, 5+10, 6+10. a) *m/z* 123, b) *m/z* 213, c) *m/z* 228, and d) *m/z* 304. The bottom images were obtained by repeating the DESI analysis on the same sample.

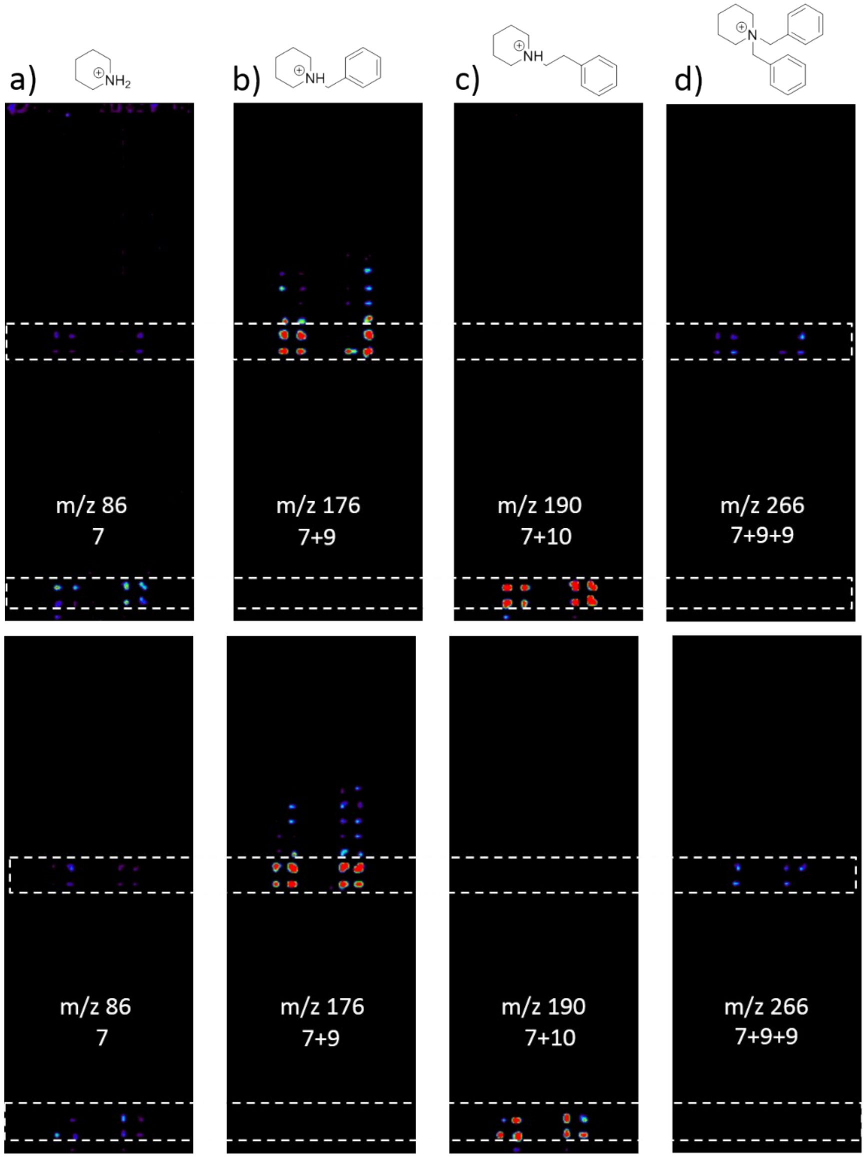

Figure S5. Selected ion images from reaction between 7+9 and 7+10. a) *m/z* 86, b) *m/z* 176, c) *m/z* 190, and d) *m/z* 266. The bottom images were obtained after repeating the DESI analysis in the same sample.

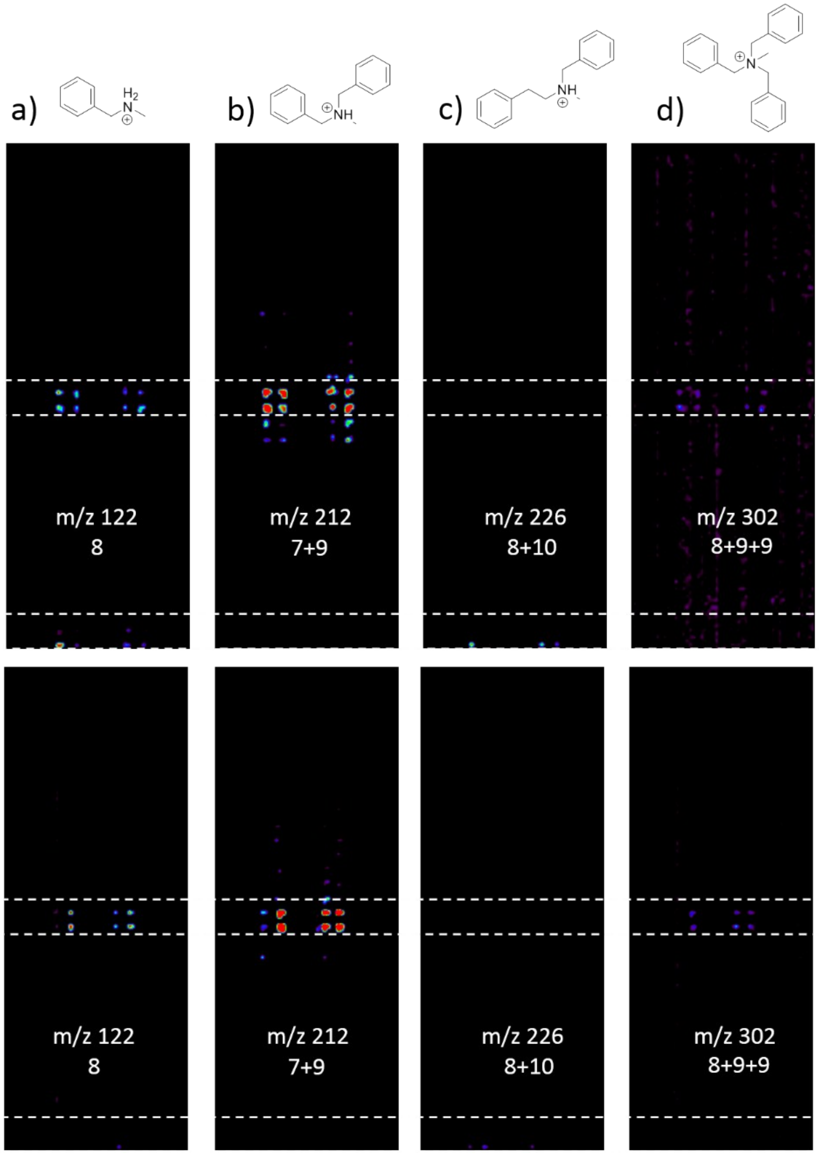

Figure S6. Selected ion images from reaction between 8+9 and 8+10. a) *m/z* 122, b) *m/z* 212, c) *m/z* 226, and d) *m/z* 302. The bottom images were obtained by repeating the DESI analysis on the same sample**.**

Table S2. Amounts of reagents spotted by the 50nL pins at the DESI plate for reaction screening for the alkylation reactions.

**Compound**	**Mass in Master Plate Well (μg)**	**Mass in DESI Spot (μg)**
Benzylamine (1)	107	0.268
Hexylamine (2)	101	0.253
Dihexylamine (3)	185	0.463
o-Anisidine (4)	123	0.308
m-Anisidine (5)	123	0.308
p-Anisidine (6)	123	0.308
Piperidine (7)	85	0.213
N-Benzylmethylamine (8)	121	0.303
Benzyl Bromide (9)	170	0.425
2-(Bromoethyl)benzene (10)	184	0.460

Table S3. Conversion rate (CR) of the 16 alkylation reactions reported at Table S1. The CR values have been calculated by dividing the average ion intensity of the product by the sum of the ion intensities of the product + reagent.

Alkylation Reaction	***Conversion Rate (CR) Product/(Product + Reagent)***
1+9	0.687
2+9	0.622
3+9	0.577

4+9	0.434
5+9	0.453
6+9	0.461
7+9	0.744
8+9	0.719
1+10	0.541
2+10	0.665
3+10	0.081
4+10	0.204
5+10	0.173
6+10	0.197
7+10	0.499
8+10	0.327

Structural characterization of alkylation reaction products

Off-line MS/MS was performed for all reactions (Tables S4 and S5). Characterization of reaction products used on-line MS/MS point-by-point analysis as described in Figure S7. This was done for four alkylation reactions (1+9, 2+9, 3+9, and 4+9). For more information on MS/MS characterization see section 6.

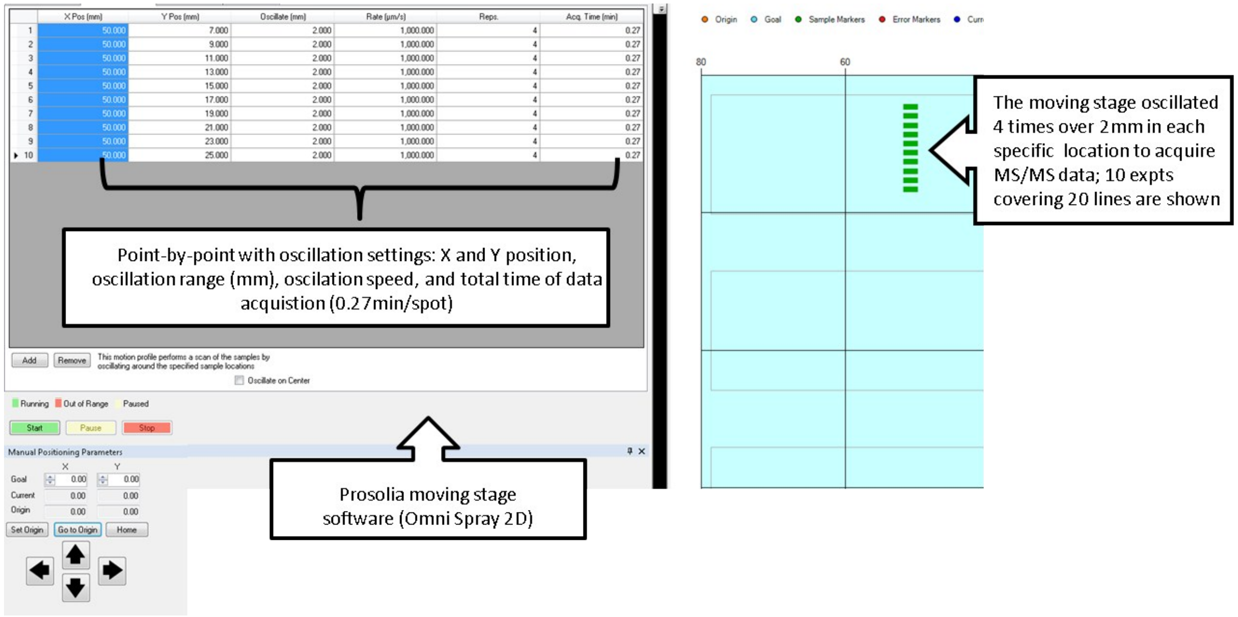

Figure S7. Printscreen of the commercial 2D DESI platform software (Prosolia Inc. Indianapolis, IN, US) with arrows and text explaining the settings used to perform on line MS/MS point-by-point analysis.

Table S4: List of product ions and relative amounts in MS/MS mass spectra of alkylation and double alkylation reaction products between 8 amines and benzyl bromide detected by DESI (see table S1).

Amine	Alkylation product *m/z*	Product ion *m/z* (relative amount)	Double Alkylation Product *m/z*	Product ion *m/z* (relative amount)
Benzylamine	198	91 (40%), 106 (20%), 181 (100%)	288	91 (15%), 181 (100%), 196 (65%)
Hexylamine	192	91 (650%)	282	91(5%), 181 (20%), 190 (20%)
Dihexylamine	276	91 (5%), 184 (15%)	366	274.2 (100%), 204.1 (15%), 181 (20%)
o-Anisidine	214	91 (65%), 123 (15%), 136 (100%)	304	136 (10%), 181 (10%), 213 (100%), 226 (10%), 272 (15%)
m-Anisidine	214	X	304	226.1 (30%), 213.1 (100%), 181 (15%), 136 (15%), 91 (5%)
p-Anisidine	214	91 (30%), 123 (85%), 136 (100%)	304	213 (100%), 226 (5%)
Piperidine	176	91 (100%), 84 (50%)	266	91 (10%), 174 (90%), 181 (100%)
N-Benzylmethylamine	212	91 (35%), 120 (35%), 181 (85%)	302	91 (10%), 134 (15%), 181 (100%), 210 (65%), 271 (10%)

Table S5: List of product ions and relative amounts in MS/MS mass spectra of alkylation and double alkylation reaction products between 8 amines and 2-bromoethylbenzene detected by DESI (see Table S1).

Amine	Alkylation product *m/z*	Product ion *m/z* (relative amount)	Elimination Product *m/z*	Product ion *m/z* (relative amount)
Benzylamine	212	91 (25%), 120 (15%), 181 (10%), 195 (100%)	210	X
Hexylamine	206	104 (100%)	204	X
Dihexylamine	290	206 (25%), 198.2 (30%), 186.2 (10%), 128.1 (10%), 105 (80%)	288	X
o-Anisidine	228	X	226	X
m-Anisidine	228	150 (25%), 136 (70%), 124 (40%), 105 (100%)	226	X
p-Anisidine	228	150 (10%), 124 (15%), 105 (100%)	226	X
Piperidine	190	105 (35%), 98 (15%), 86 (5%)	188	X
N-Benzylmethylamine	226	X	224	X

3. Suzuki Cross Coupling

Stock solutions of **11** (0.4 M in ethanol, **12** (0.4 M in ethanol), XphosPD G3 (0.04 M in ethanol), and base (0.8 M in ethanol, except sodium methoxide which was 0.5 M in ethanol) were prepared and dispensed in equal volumes into a 384 master plate resulting in a final volume of 20 µl in each well. In some control experiments different combinations of reagents were used. Additionally, 100 µl of a prepared solution of Rhodamine B (25 mg/ml in acetonitrile acidified with ~10 µl of 4M HCl in dioxane) was also added to the indicated wells (Figure S8). A pin tool fitted with 6nL pins was used to transfer solution from the master plate onto suitable DESI-MS substrates. Each sample well in the master plate was transferred in quadruplicate resulting in 1536 density on the DESI-MS substrate. The freshly prepared membrane substrate was transferred to a homebuilt DESI source for analysis. The analysis speed was 6,250 µm/sec resulting in a reaction rate of 1,284 reactions/hr

Table S6 List of components on master plate for Suzuki reaction

**11**	4-hydroxyphenylboronic acid	**15**	Sodium ethoxide
**12**	3-bromopyridine	**16**	Potassium ethoxide
**13**	RXN Product	**17**	Sodium methoxide
**14**	XphosPD G3	**18**	Potassium methoxide

4. Ultrahigh density spotting experiment

	1	2	3	4	5
A	RhB	18	12+18	18	RhB
B	RhB	17	12+14	17	RhB
C	RhB	16	11+18	16	RhB
D	RhB	15	11+14	15	RhB
E	RhB	18	12+17	18	RhB
F	RhB	17	12+14	17	RhB
G	RhB	16	11+14	16	RhB
H	RhB	15	11+17	15	RhB
I	RhB	18	12+16	18	RhB
J	RhB	17	11+16	17	RhB
K	RhB	16	12+15	16	RhB
L	RhB	15	12+14	15	RhB
M	RhB	18	11+15	18	RhB
N	RhB	17	11+14	17	RhB
O	RhB	16	12	16	RhB
P	RhB	15	11	15	RhB

Figure S8: Reaction solution position in master plate for experiment 1. Columns 2 and 4 also contain **11**, **12**, and **14**.

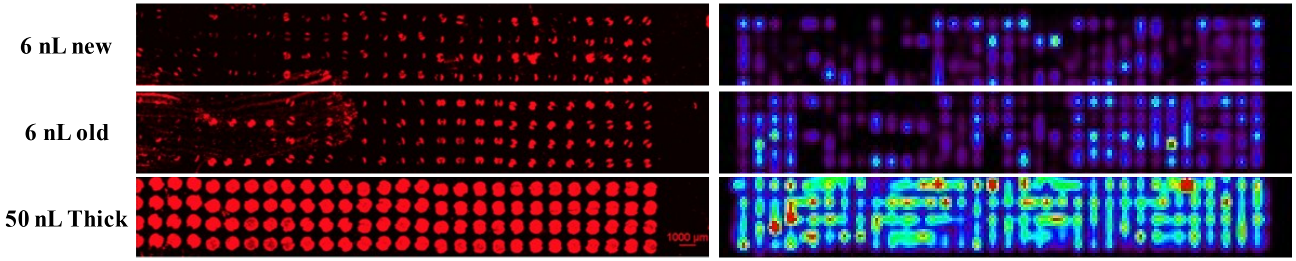

Figure S9. Comparison of fluorescence (left) and DESI (right) screening of the same 6,144-well microtiter plate loaded with alkyl amine and alkyl bromide mixtures using the different pin types indicated.

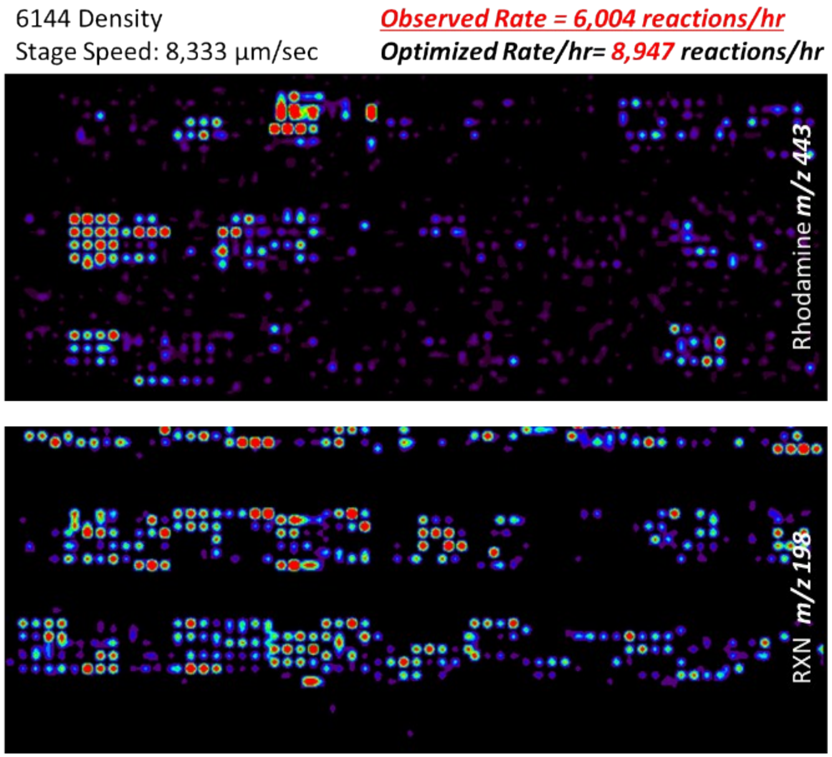

Figure S10. Analysis of rhodamine B and the reaction between benzylamine **1** and benzyl bromide **9** at 6,144 density using an analysis speed of 8,333 µm/sec. Optimized rate refers to conditions in which the line turnaround time is minimized.

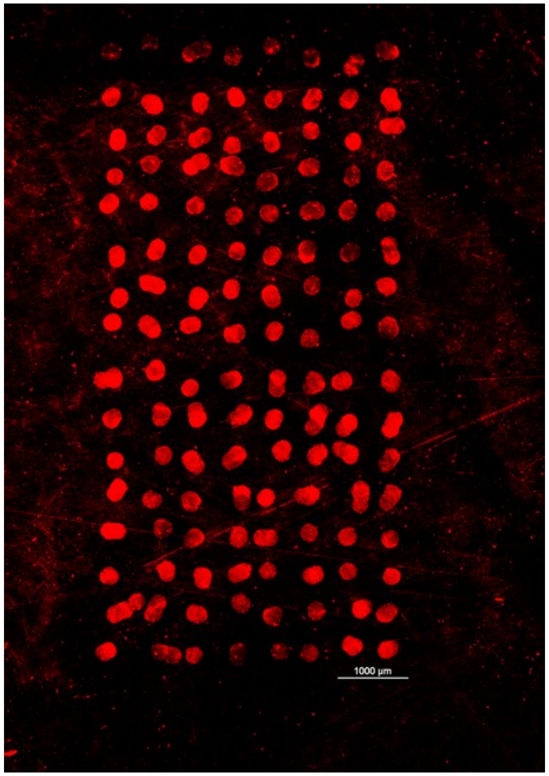

Figure S11. Fluorescence imaging (spot density: 24,457 / per standard well plate; Solvent: H2O:ACN (7:3); Membrane: Millipore PTFE (3 µm); using 4 nL pins). The fluorescence image demonstrates the capability to spot at this density. The DESI image (not shown) has lower resolution.

5. Typical MS spectra

Figure S12 shows typical MS spectra of four N-alkylation reactions. Note the marked signals corresponding to the desired product, dibenzylamine,

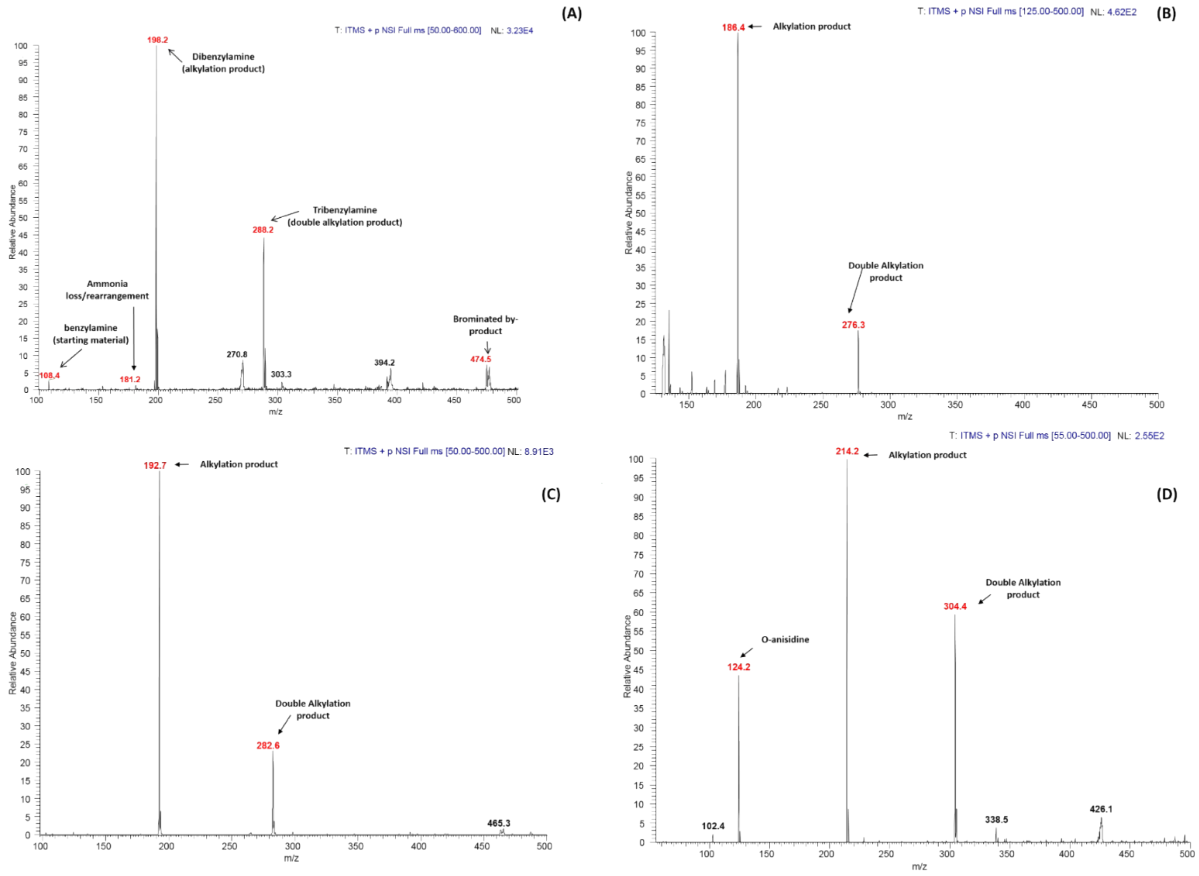

and two by-products.

Figure S12. (A) Full mass scan (m/z 50-500; offline data, background of PTFE surface subtracted) of selected N-amine alkylations. (A) reaction of benzylamine with benzyl bromide. The starting material, reaction product (dibenzylamine) and by-products are indicated; (B) reaction of dihexylamine with benzyl bromide; (C) reaction of hexylamine with benzyl bromide; (D) reaction of o-anisidine with benzyl bromide.

6. Product identification by MS/MS

For the benzylamine with benzyl bromide reaction, mass spectra of individual spots give indications as to product and by-product formation but MS/MS data are needed for confirmation. The benzylamine/benzyl bromide reaction is a case in point given the high background in the mass spectrometer. The identity of the product as dibenzylamine is demonstrated by MS/MS (Fig. 4).

The prominent ion at *m/z* 288 in the reaction mixture is of interest as a likely by-product, with molecular weight 287 Da. Again off-line MS/MS was applied and fragmentation of $[M + H]^+$ occurs by ammonia loss (Fig S13), a reaction which parallels the rearrangement of dibenzylamine. It is therefore assigned as a true by-product, not a different ionized form of the product. To confirm the by-product structure, on-line MS/MS was also performed (Fig. S14). This was done by re-scanning the array and investigating those pixels which were positive for *m/z* 288.

The fragmentation pattern observed in Figs. S13 and S14 suggests the structure tribenzylamine for the byproduct. To establish this, the reaction between **1** and **9** was run in bulk and the by-product was purified by recrystallization and silica gel column chromatography. The purified product of *m/z* 288 fragments in the same manner as the product from the DESI reaction. The NMR of *m/z* 288 confirms the structure of tribenzylamine (Figure S16). The fragmentation scheme is shown in Scheme S3.

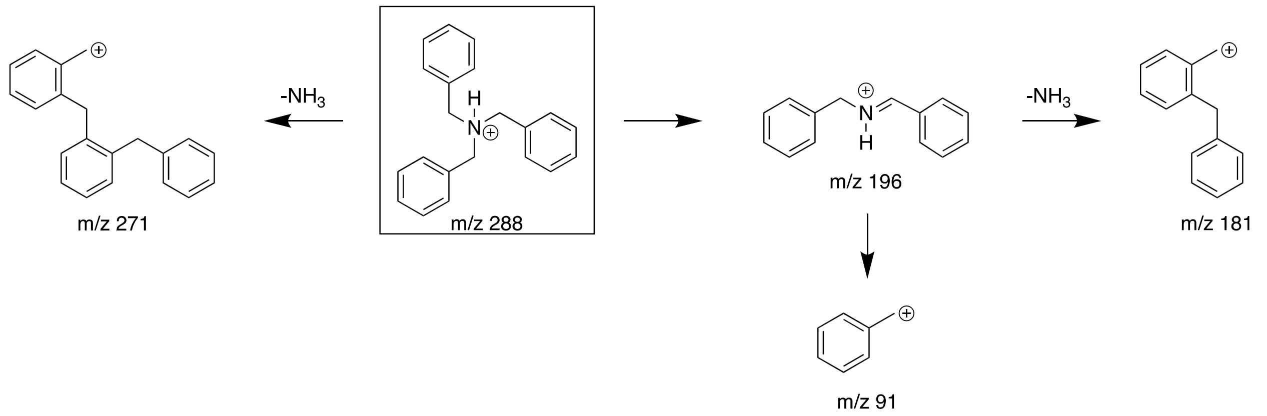

Scheme S3. Fragmentation of by-product molecular weight 287, *m/z* 288, showing formal elimination of toluene to give *m/z* 196.

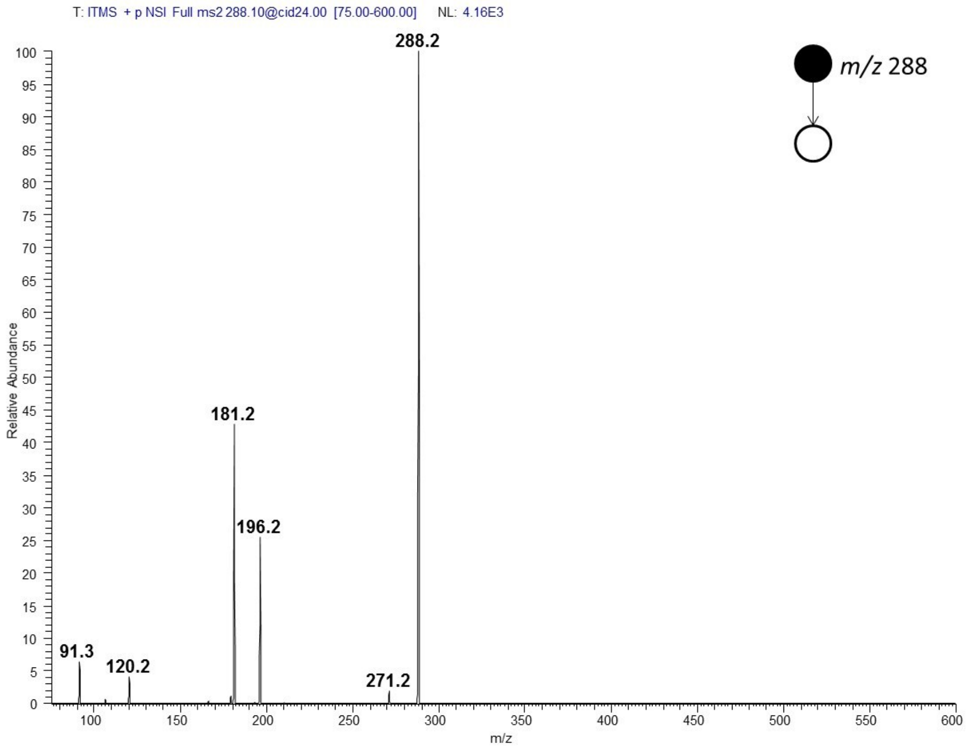

Fig S13. Off-line product ion MS/MS spectrum of m/z 288.

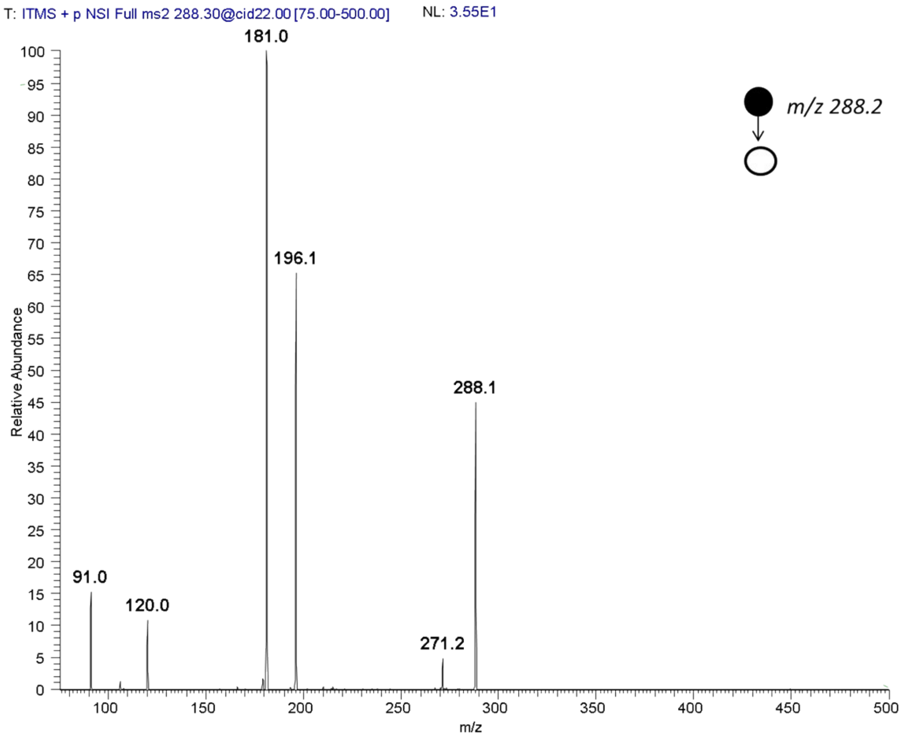

Fig S14. On-line product ion MS/MS spectrum of m/z 288

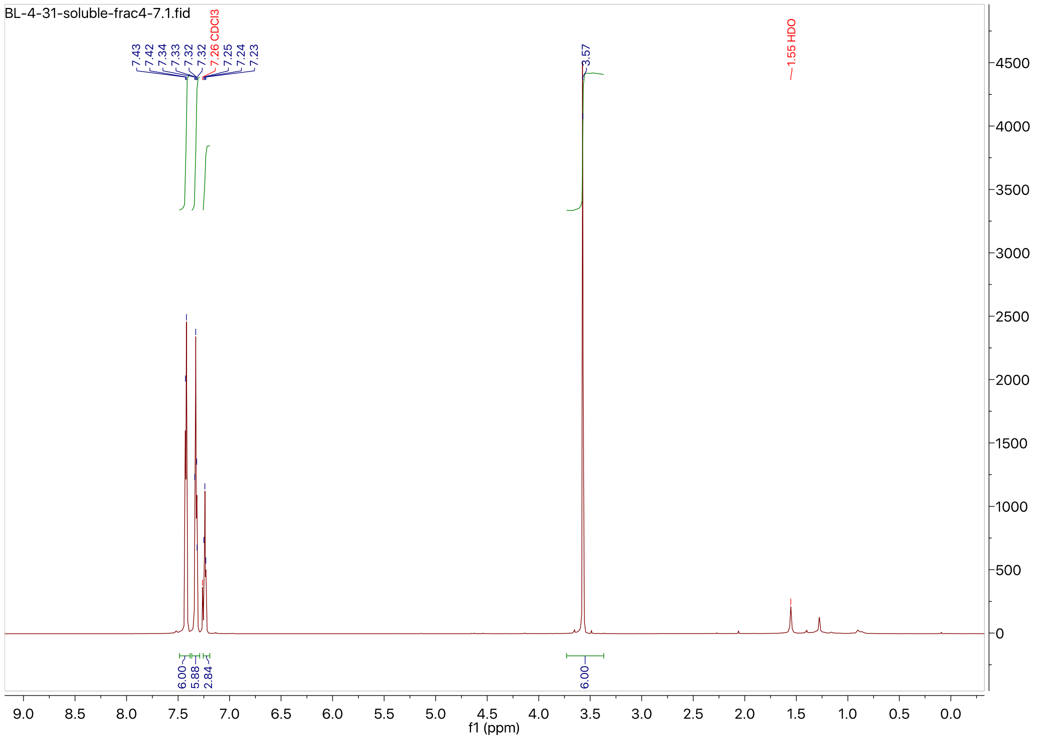

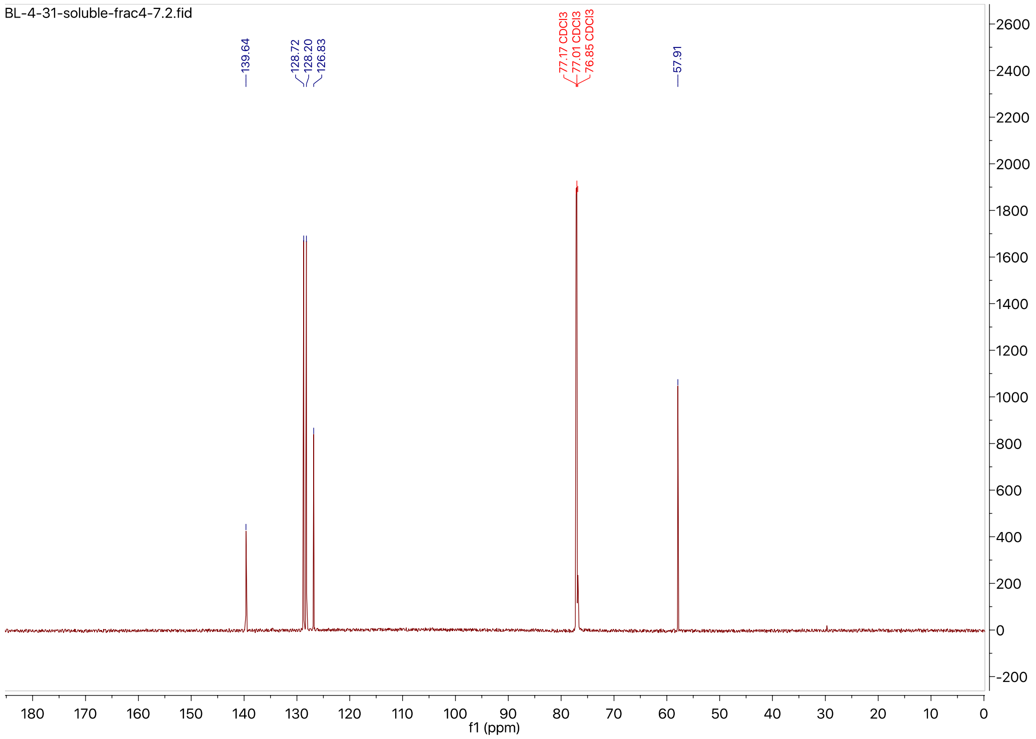

Fig S15. NMR of purified *m/z* 288 after bulk synthesis.

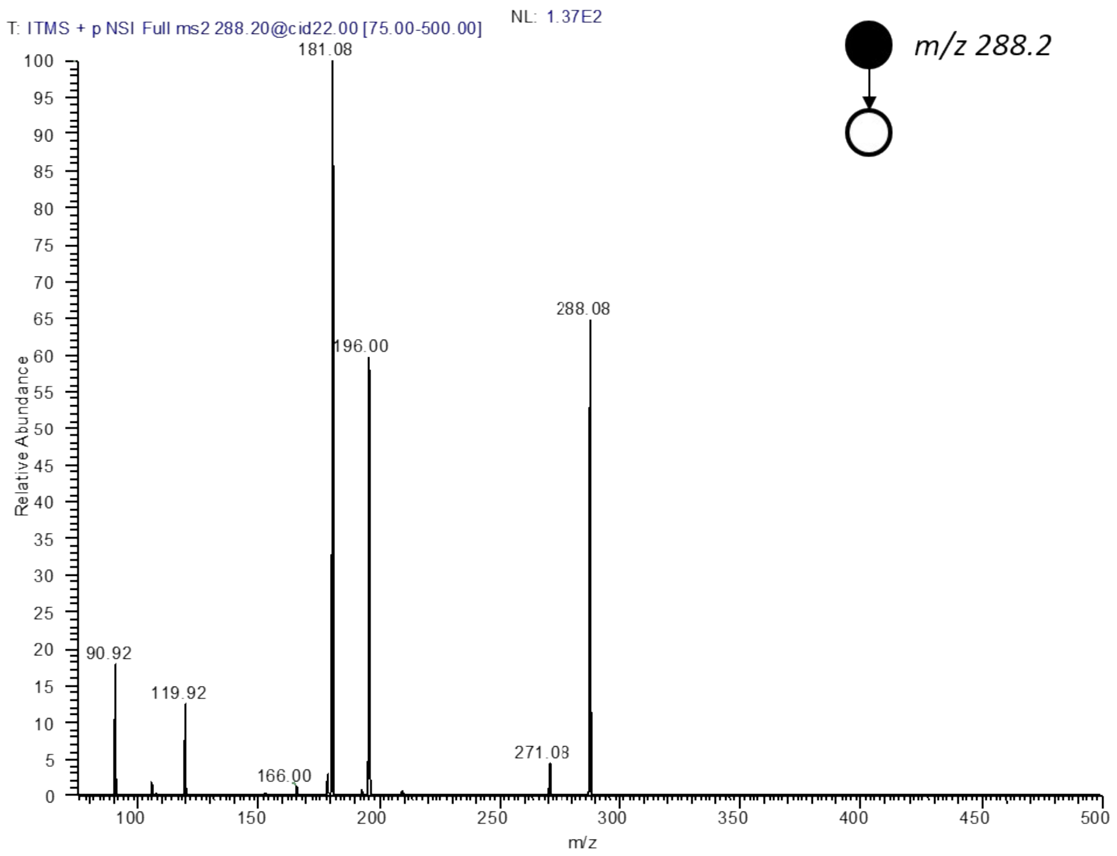

Fig S16. MS/MS of purified *m/z* 288 after bulk synthesis

Another feature of the mass spectrum of the reaction mixture of benzylamine and benzyl bromide is the ion pair *m/z* 474/476. This species appears to be brominated from the isotope pattern and this is supported by Figure S17A and B which shows their individual MS/MS spectra. The low signal intensity caused these spectra to be recorded as the sum of the spectra of a row of pixels. The data were recorded on-line but the low signals meant that 2 Da windows were used for the precursor ion selection and to record product ion spectra. DESI-MS imaging figures show online MS/MS data for individual pixels of two precursor ions in the benzylamine/benzyl bromide reaction mixture (Fig. S17). The upper line of spots depicts MS/MS of 476 and the bottom line of spots data for MS/MS of 474. The brominated species lose bromine to give the protonated dimer.

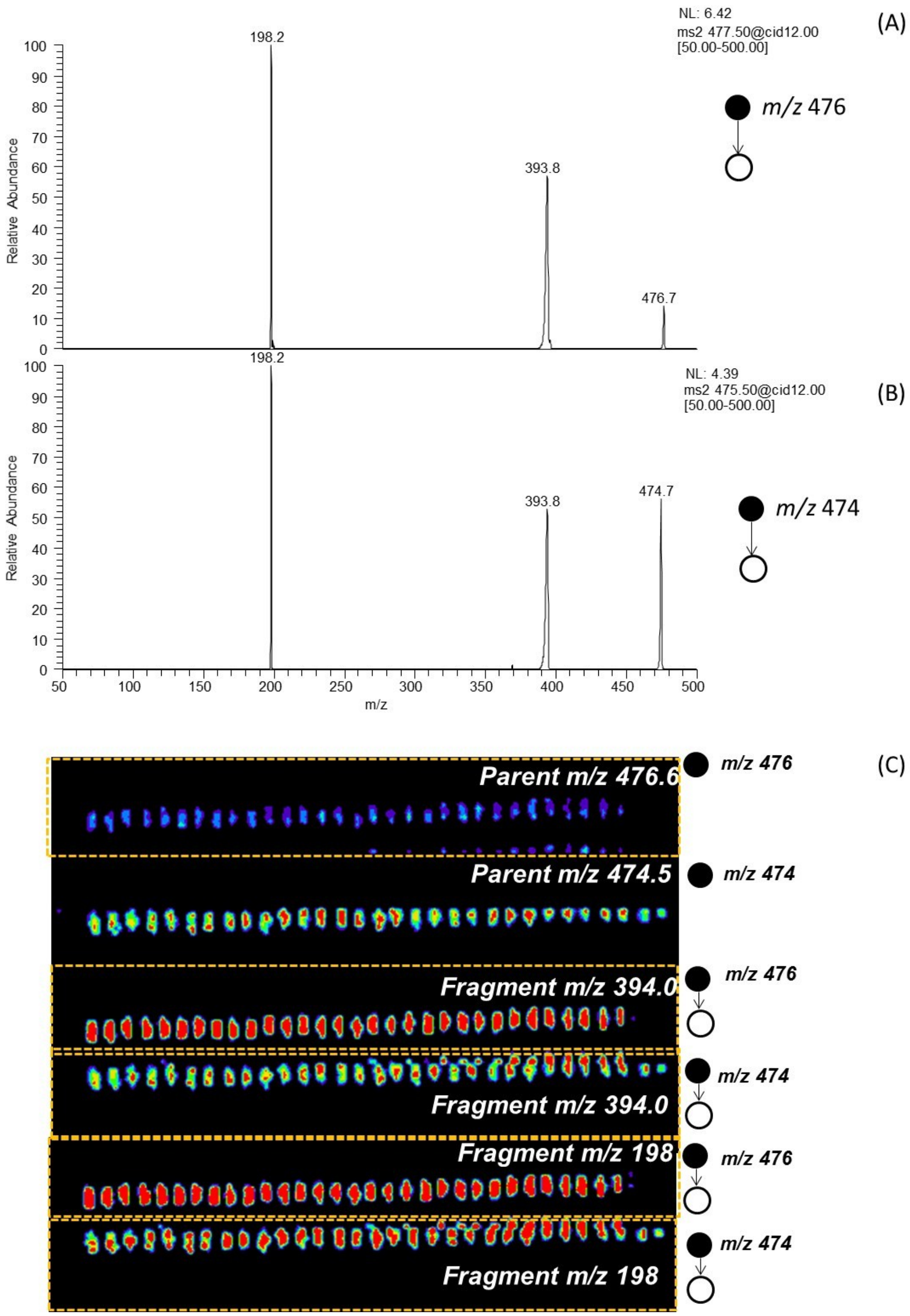

Figure S17. (A) and (B) On-line MS/MS spectra for the reaction products of benzylamine and benzyl bromide, monoisomeric masses *m/z* 476 and *m/z* 474 using collision energy 12 (LTQ XL, Thermo Scientific); (C) DESI-MS imaging of two lines of spots on PTFE surface. The upper line has been scanned in MS/MS mode for the products of *m/z* 476 and the bottom line in MS/MS mode for the *m/z* 474. The first two lines show the precursor ions under conditions were it is being fragmented (hence the signals are weak) The four product ion images show *m/z* 476 and 474 which give *m/z* 394 and *m/z* 198. This confirms the bromination but the structure of this product has not been determined.

7. On-line MS/MS by re-analyzing selected pixels

This procedure was used to record on-line data on m/z 288 as described in sec. 5, above. The high quality of the information obtained on a second scan may be inferred from the data shown for two reactions in Fig. S19

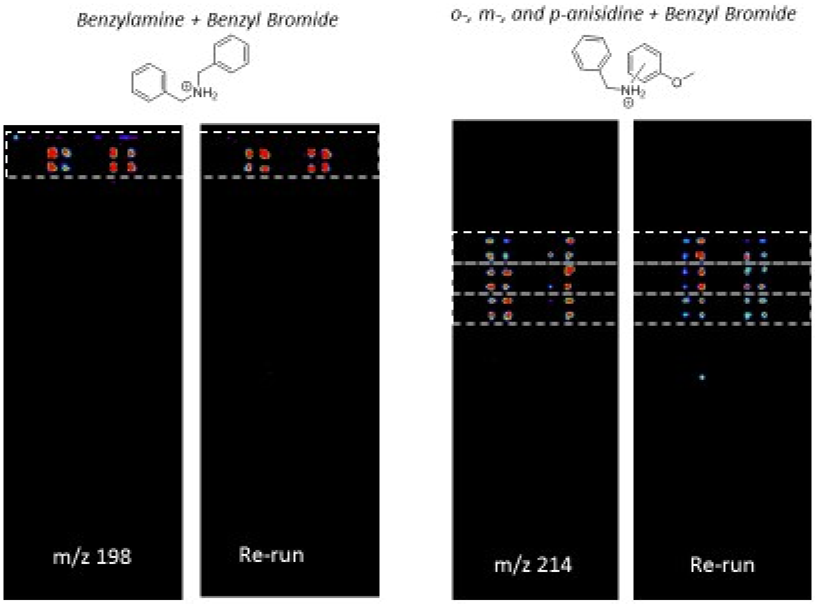

Figure S18. Re-analysis of reactions by DESI (left) benzyl bromide and benzylamine (right) benzyl bromide and the o-, m- and p-methoxyanilines

8. Structural elucidation of reaction products by MS^n

For the reaction of dihexylamine + benzyl bromide, MS^3 and MS^5 data have been acquired for a more detailed study of the alkylation (*m/z* 276) and double alkylation (*m/z* 366) products, respectively. The number of steps in an MS^n experiment depends on the ion signal of the isolated fragments and the ease of their further fragmentation. Fig. S20 shows the annotated MS^3 of *m/z* 276 and Fig. S21 the MS^5 of *m/z* 366.

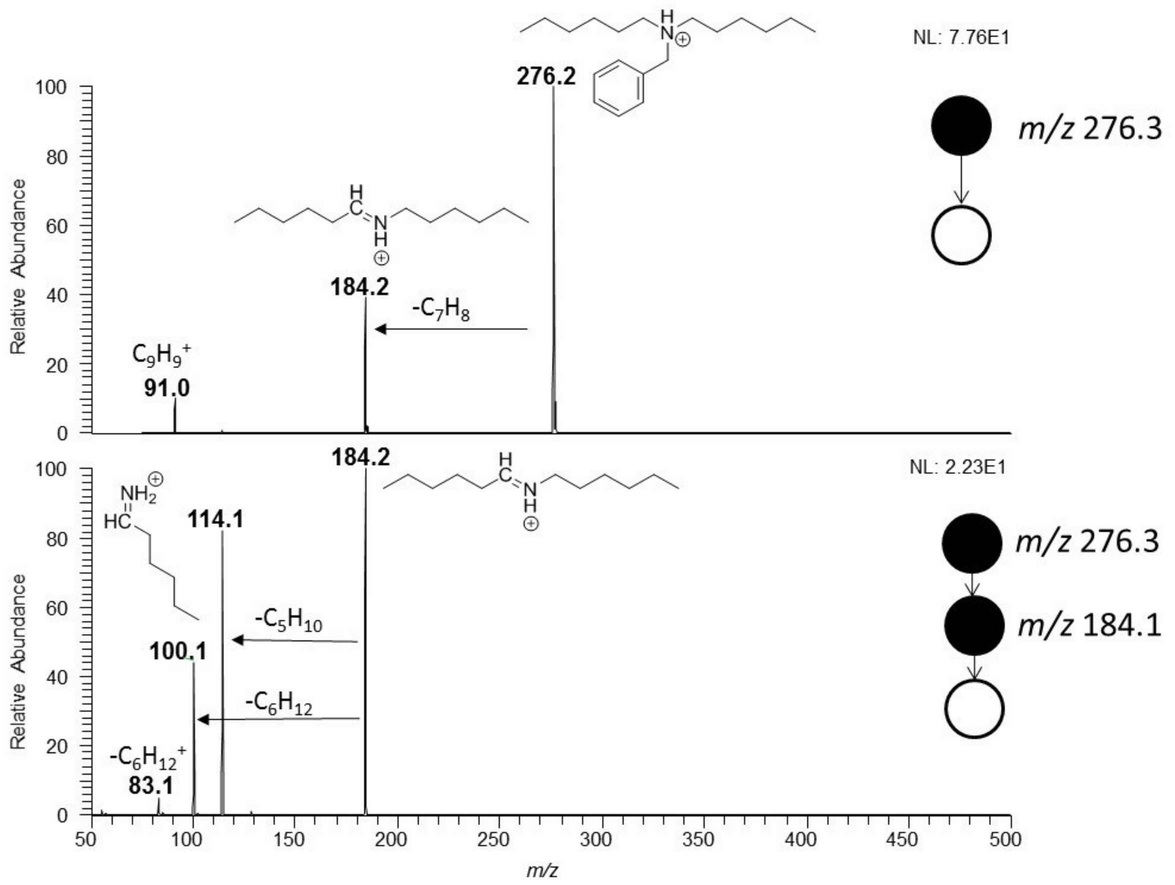

Figure S19. Annotated MS[3] of the alkylation product of dihexylamine + benzyl bromide, *m/z* 276. MS[2] shows neutral loss of an alkene (C_7H_8) of *m/z* 184, which has been further isolated for MS[3]. In the MS[3] spectrum, additional neutral losses of alkenes are observed as well as formation of an amine (of *m/z* 100) and the tolyl/tropylium ion (*m/z* 91).

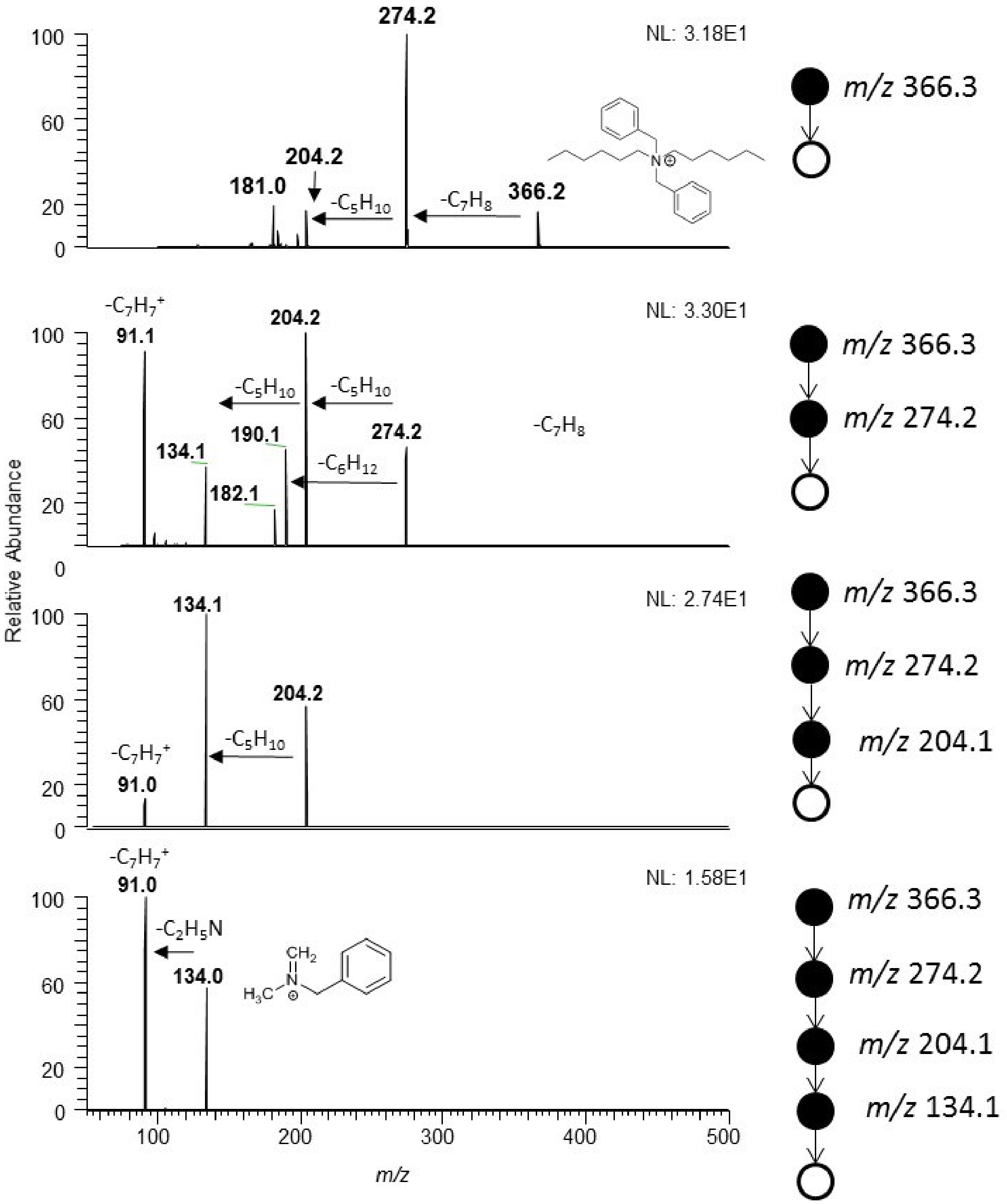

Figure S20. Annotated MS[5] of dihexylamine + benzyl bromide double alkylation product, *m/z* 366. MS[2] yielded *m/z* 274 as the most intense fragment due the loss of alkene (C_7H_8). In the MS[3], *m/z* 274 yielded *m/z* 204 due to another alkene loss (C_5H_{10}) and formation of the tolyl/tropylium ion (*m/z* 91). MS[4] of *m/z* 204 showed another alkene loss (C_5H_{10}) to yield *m/z* 134, which was intense enough to be isolated to study its fragmentation in the MS[5] spectrum.
